# Potential Pitfalls and Solutions for Use of Fluorescent Fusion Proteins to Study the Lysosome

**DOI:** 10.1371/journal.pone.0088893

**Published:** 2014-02-21

**Authors:** Ling Huang, Douglas Pike, David E. Sleat, Vikas Nanda, Peter Lobel

**Affiliations:** 1 . Center for Advanced Biotechnology and Medicine, Rutgers University, Piscataway, New Jersey, United States of America; 2 Department of Pharmacology Robert Wood Johnson Medical School, Rutgers University, Piscataway, New Jersey, United States of America; 3 Department of Biochemistry and Molecular Biology, Robert Wood Johnson Medical School, Rutgers University, Piscataway, New Jersey, United States of America; The University of New South Wales, Australia

## Abstract

Use of fusion protein tags to investigate lysosomal proteins can be complicated by the acidic, protease-rich environment of the lysosome. Potential artifacts include degradation or release of the tag and acid quenching of fluorescence. Tagging can also affect protein folding, glycosylation and/or trafficking. To specifically investigate the use of fluorescent tags to reveal lysosomal localization, we tested mCherry derivatives as C-terminal tags for Niemann-Pick disease type C protein 2 (NPC2), a luminal lysosomal protein. Full-length mCherry was released from the NPC2 chimera while deletion of the 11 N-terminal residues of mCherry generated a cleavage-resistant (cr) fluorescent variant. Insertion of proline linkers between NPC2 and crmCherry had little effect while Gly-Ser linkers promoted cleavage. The NPC2-crmCherry fusion was targeted to the lysosome and restored function in NPC2-deficient cells. Fusion of crmCherry to known and candidate lysosomal proteins revealed that the linkers had different effects on lysosomal localization. Direct fusion of crmCherry impaired mannose 6-phosphorylation and lysosomal targeting of the lysosomal protease tripeptidyl peptidase I (TPP1), while insertion of linkers corrected the defects. Molecular modeling suggested structural bases for the effects of different linkers on NPC2 and TPP1 fusion proteins. While mCherry fusion proteins can be useful tools for studying the lysosome and related organelles, our findings underscore the potential artifacts associated with such applications.

## Introduction

Lysosomes are membrane-delimited, acidic organelles present in essentially all eukaryotic cells [1]. This compartment contains over 70 hydrolytic enzymes [2], [3] that function to degrade biological macromolecules taken up by endocytosis, phagocytosis and autophagy. There is considerable interest in studying the role of lysosomes in basic biological processes and in diseases including lysosomal storage disorders, neurodegeneration, cancer, and inflammation.

Fluorescent fusion proteins are invaluable tools in biomedical research but there are specific considerations in their use to study the lysosome. The lumen of the lysosome is an acidic environment that is rich in multiple proteases thus it is essential that fluorescent tags are resistant to both proteolytic degradation and acid quenching [4]. Monomeric red fluorescent protein (mRFP) and its derivative mCherry have been widely used to study autophagic flux due to their persistent fluorescence in the lysosome [4]–[7]. However, previous studies have noted that, for some fusion proteins targeted to the lysosome, the reporter tag may be cleaved from the parent protein [8], [9]. In some cases, this problem can be addressed by reducing lysosomal protease activity through use of inhibitors or mutant cells deficient in lysosomal proteases [8], though this is not ideal as it may introduce artifacts. Fluorescent tags used for the study of localization must not perturb protein targeting.

In this study, we used a combination of structure-based protein design, protein engineering, and empirical testing to evaluate the integrity of fluorescent tags fused to lysosomal proteins and the effect of construct design on the targeting of these proteins. We have focused on Niemann-Pick disease type C protein 2 (NPC2), a lysosomal cholesterol binding protein [10] whose high-resolution structure has been determined [11], [12]. We find that construct design, particularly with respect to the linker region connecting the protein of interest to the C-terminal fluorescent tag, can have a dramatic effect on the integrity of the fusion protein. We have expanded this analysis to study the effect of the linker region on fusions between mCherry and the lysosomal protease tripeptidyl peptidase I (TPP1) and candidate lysosomal proteins arylsulfatase K (ARSK), alpha-L-2 fucosidase (FUCA2) and lactoperoxidase (LPO). The nature of the linker used had significant effects on lysosomal targeting, possibly by interfering with protein folding, posttranslational modification, and/or recognition by targeting machinery. However, we demonstrate that aberrant localization can be ameliorated with an appropriate choice of linker.

## Results and Discussion

### Integrity of NPC2-mCherry Fusion Proteins Varies with Linker Sequence

In the course of studies investigating the molecular basis for lysosomal cholesterol efflux, we sought to develop a fluorescent NPC2 fusion protein that retained function and integrity in the lysosome. mCherry was chosen primarily due to its resistance to acid quenching [13]. Additionally, it is a true monomer [14], which is important as previous studies have demonstrated that oligomeric GFP-like proteins commonly form aggregates, especially when highly expressed [13], [15].

**Figure 1:**
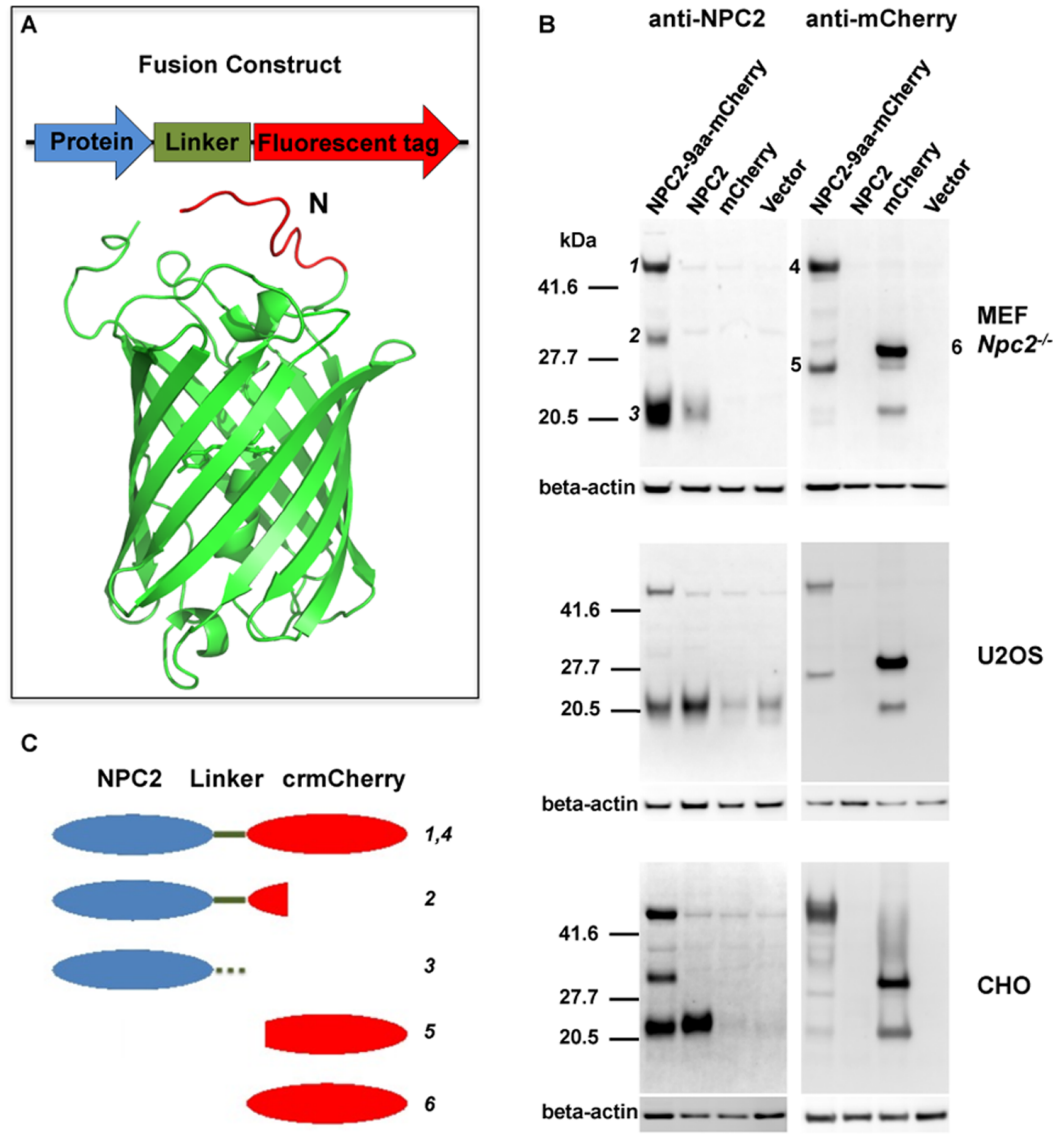
Stability of NPC2-mCherry fusion proteins. A) Design of fusion proteins and schematic of mCherry. Fluorescent tags were engineered to the C-termini of proteins of interest. The N-terminal 11 amino acid region of mCherry (MVSKGEEDNMA) was deleted to create crmCherry. In the mCherry structure shown, residues 1–11 are shown in red. B) *Npc2^−/−^* MEFs, U2OS, and CHO cells were transiently transfected with constructs expressing mCherry alone, NPC2 alone or an NPC2-mCherry fusion with a 9aa linker sequence (RARDPPVAT). After 48 h, cell lysates were analyzed by immunoblotting for NPC2 or mCherry. β-actin was used as a loading control. C) Schematic illustrating the indicated numbered bands shown in Panel B.

An initial fusion protein was constructed with the C-terminal Ser of mouse NPC2 linked via the peptide RARDPPVAT, which is encoded by the plasmid multiple cloning site, to the N-terminal Met of mCherry (Fig. 1A, NPC2-9aa-mCherry). There was significant proteolytic cleavage of this chimera expressed in *Npc2^−/−^* immortalized mouse embryonic fibroblasts (MEFs), the human osteosarcoma cell line U2OS, and Chinese Hamster Ovary (CHO) cells (Fig. 1B). All three cell types showed a similar pattern. The intact 49 kDa fusion protein is detected with both α-NPC2 and α-mCherry antibodies (Bands 1 and 4, Fig. 1B). In the α-NPC2 blot, a 20 kDa species is the major immunoreactive species observed in cells transfected with the fusion protein (Band 3, Fig 1B) and this co-migrates with the transfected murine NPC2. This indicates significant cleavage near the linker region, resulting in separation of NPC2 and mCherry. There is another degradation product of 32 kDa (Band 2, Fig. 1B), which likely represents NPC2 with the linker and an N-terminal portion of mCherry. When probing for mCherry, a 27 kDa fusion protein degradation product (Band 5, Fig. 1B) migrates slightly faster than the 29 kDa full-length mCherry (Band 6, Fig. 1B).

**Figure 2:**
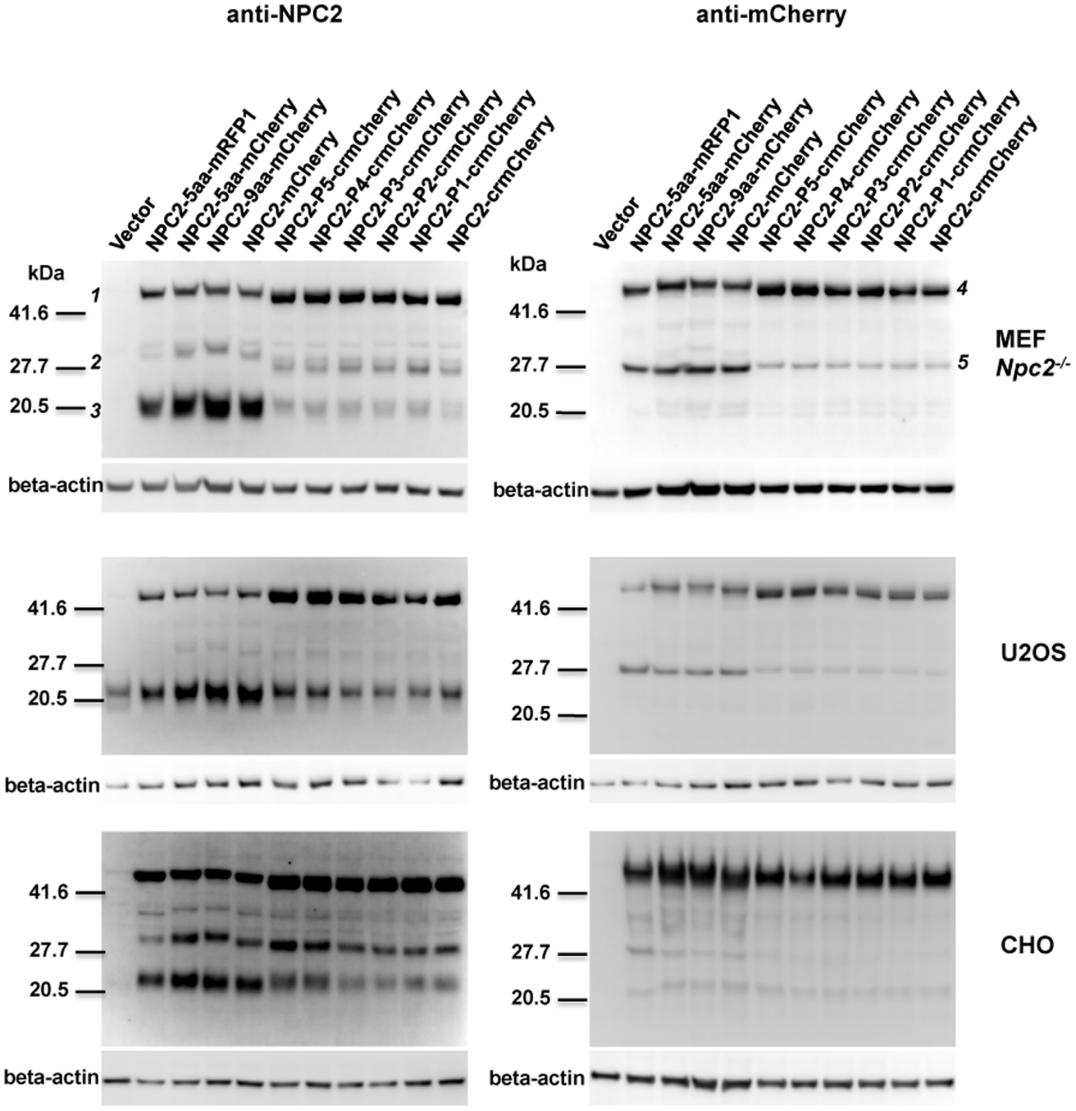
Effect of linker region on stability of NPC2 fusion proteins. *Npc2^−/−^* MEFs, U2OS, and CHO cells were transiently transfected with a vector control or indicated fusion constructs. Linker sequences are as described in Fig. 1 legend, and below: 5aa: (PPVAT), P5: (PPPPP), P4: (PPPP), P3: (PPP), P2: (PP), P1: (P). After 48 h, cell lysates were analyzed by immunoblotting as in Fig. 1.

**Figure 3:**
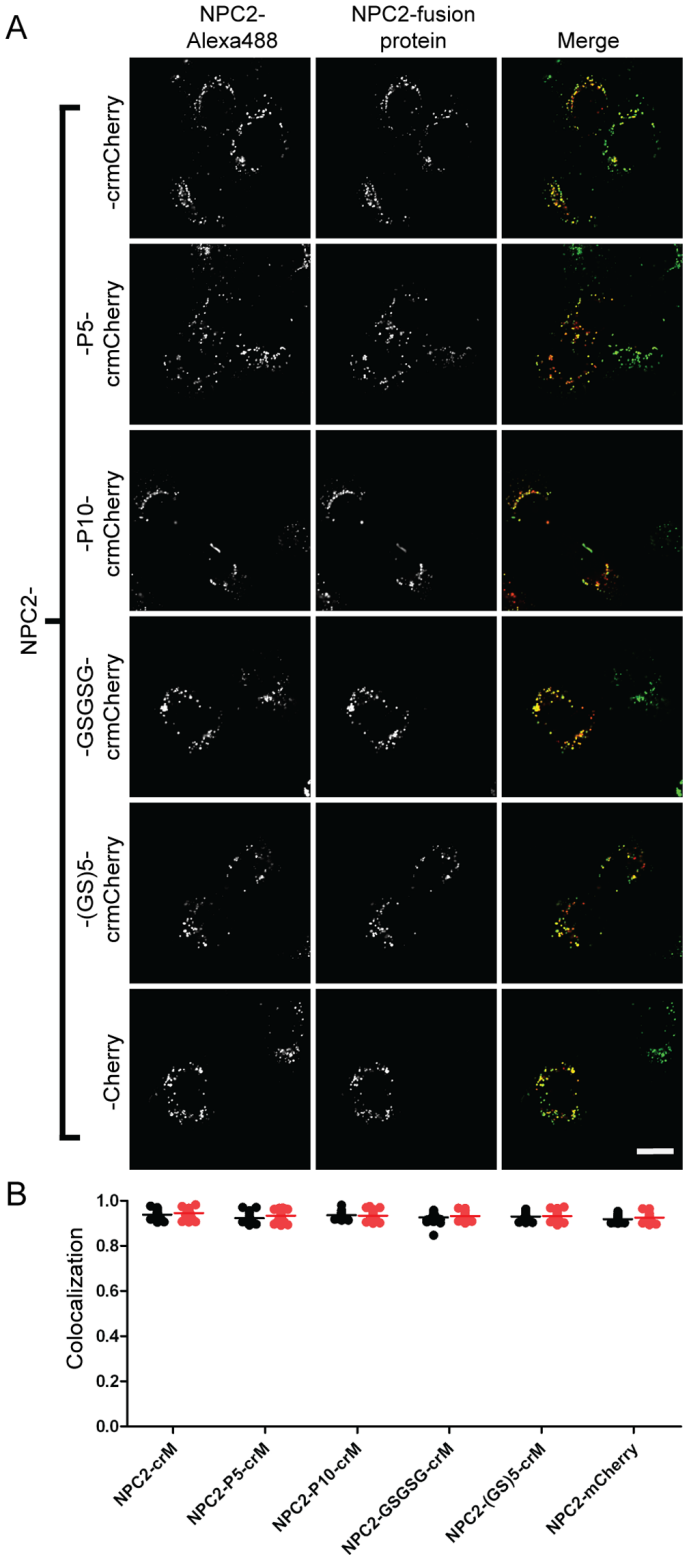
Subcellular localization of NPC2-crmCherry fusion proteins. A) U2OS cells were transiently transfected with indicated fusion constructs. Linker sequences are as described in Fig. 2 legend and below: P10: (PPPPPPPPPP); (GS)5, (GSGSGSGSGS). Endocytosed Alexa488-NPC2 is used as a lysosomal standard. The scale bar (white) in the bottom right corner represents 20 µm. (B) Quantitative co-localization analysis. Mander’s colocalization coefficients (tM) were plotted for individual fields of cells. Black dots (tM1) represent Mander’s coefficients for the fraction of pixels containing red signal from crmCherry/mCherry that also contained signal from green-NPC2, while red dots (tM2) represent Mander’s coefficients for the fraction of lysosomes (marked by green-NPC2) that also contain red fluorescent protein chimera. Each point represents analysis of a single field containing 1–4 transfected cells. The number of cells analyzed for each fusion construct are as follows: NPC2-crM (22), NPC2-P5-crM (45), NPC2-P10-crM (29), NPC2-GSGSG-crM (28), NPC2-(GS)5-crM (39), NPC2-mCherry (34).

Since significant cleavage occurred between NPC2 and mCherry, the linker and N-terminal region of mCherry were targeted for protein engineering in an attempt to improve stability. The first three residues of mCherry are not resolved in its crystal structure [16], implying that they are dynamic. The following eight residues lack regular secondary structure (Fig. 1A, marked red). We therefore suspected that the N-terminal 11 amino acids would be susceptible to proteolysis and thus removed them from the full-length mCherry, with the deletion mutant designated crmCherry. We then used different linkers (listed in legends to Figs. 2 and 3) to connect NPC2 to the C-terminus of crmCherry, mCherry, and mRFP1.

Constructs were transfected into *Npc2^−/−^* MEFs, U2OS, and CHO cells to assess stability of the fusion proteins (Fig. 2). In all cell types, the crmCherry fusion proteins exhibited significantly less cleavage compared to mCherry or RFP1 fusion proteins. This is consistent with the idea that the flexible 11-amino acid N-terminus of mCherry is especially vulnerable to cleavage by lysosomal endoproteases. Insertion of one to five prolines between crmCherry and NPC2 had little effect. A slight difference in the electrophoretic mobility of the full-length fusion proteins among the different constructs is visible (Fig. 2, Bands 1 and 4). This reflects differences in the linker and the N-terminal region of mCherry. Band 2 shows corresponding differences in mobility to those found in the full-length fusion proteins, indicating that the proteins indicated by Band 2 represent full length NPC2 with different linkers and a small section of the fluorescent tag. Band 5 is of similar mobility in all constructs, indicating that it does not contain the variable-length linker regions or the N-terminal region of the fluorescent tags. This band therefore represents proteins that contain most of the C-terminal portion of mCherry.

To further diminish proteolytic release of crmCherry from NPC2, we explored the use of other linkers in the fusion protein, examining the role of both length and flexibility. Proline is the most conformationally constrained amino acid, glycine is the most flexible, and serine has a relatively small, hydrophilic side chain with intermediate flexibility. The integrity of chimeras containing proline linkers of variable length was similar to that of the direct fusion with crmCherry (Fig. S1). In contrast, chimeras with five or ten residue Gly-Ser linkers were more susceptible to cleavage (Fig. S1). The reduced cleavage of the polyproline linker may be due to the specificity of lysosomal endopeptidases or the reduced flexibility of the imino acid peptide bond, this may reflect flexibility/accessibility. Regardless, the cleavage of the full-length NPC2-mCherry chimeras and the crmCherry fusion proteins containing Gly-Ser linkers underscores how domain separation with susceptible residues can result in increased lysosomal proteolysis.

### Lysosomal Targeting and Biological Activity of NPC2-crmCherry

We conducted further analyses to determine if the fluorescent tag interfered with NPC2 localization and/or function. We have previously shown that purified recombinant human (rh) NPC2 can be delivered to the lysosome of cultured cells through mannose 6-phosphate (M6P) receptor mediated endocytosis [17]. Therefore, we conjugated purified rhNPC2 to Alexa Fluor 488 (green-NPC2) and used the endocytosed labeled protein as a lysosomal marker. Live cells were analyzed by confocal microscopy and colocalization estimated using Mander’s colocalization coefficient (tM), which can vary from one (complete colocalization) to zero (no colocalization) [18]. Two coefficients were analyzed. The fraction of pixels containing signal from the red fluorescent fusion proteins that also contained signal from green-NPC2 is given by tM1 and represents the fraction of NPC2-fusion protein that is associated with the lysosome. Conversely, tM2 represents the fraction of lysosomes (marked by green-NPC2) that also contain red fluorescent protein chimeras. Almost complete colocalization (tM1 and tM2>0.9) was obtained when different NPC2-mCherry variants were transiently expressed in U2OS cells (Fig. 3) and similar colocalization was observed in transiently transfected MEFs and CHO cells (Fig. S2). Thus, the fusion protein does not interfere with lysosomal targeting of NPC2. It is worth noting that for variants where the mCherry was cleaved from the chimera, there was colocalization of the mCherry fluorescence with the lysosomal marker, demonstrating that proteolysis and release of the fluorescent tag is occurring within the lysosome.

**Figure 4:**
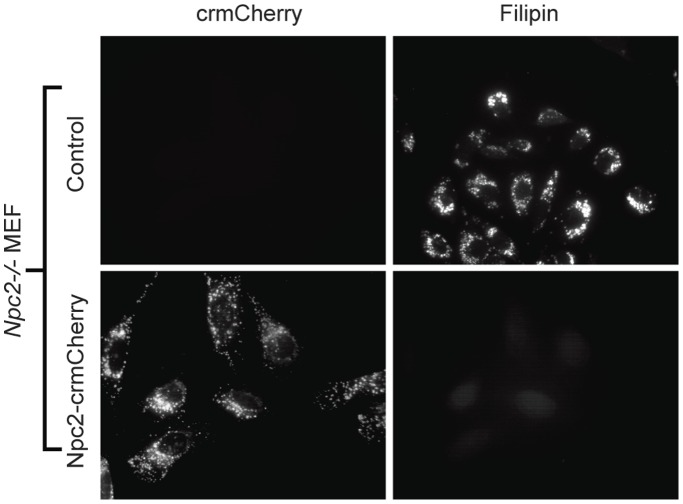
Function of the NPC2-crmCherry fusion protein. *Npc2^−/−^* MEF cells were stably transfected with the NPC2-crmCherry fusion construct and compared to non-transfected control. Cholesterol accumulation was visualized by staining with filipin.

NPC2 functions to facilitate egress of cholesterol from the lysosome and deficiencies in NPC2 result in lysosomal accumulation of unesterified cholesterol that can be visualized by filipin staining [10]. We tested the functionality of NPC2-crmCherry in stably transfected *Npc2^−/−^* MEFs. This fusion construct completely rescued the cholesterol storage phenotype (Fig. 4), indicating that the fusion protein tag does not interfere with cholesterol binding or other downstream functions of NPC2.

### Linker Sequence Affects Lysosomal Targeting of TPP1-crmCherry Fusion Protein

**Figure 5:**
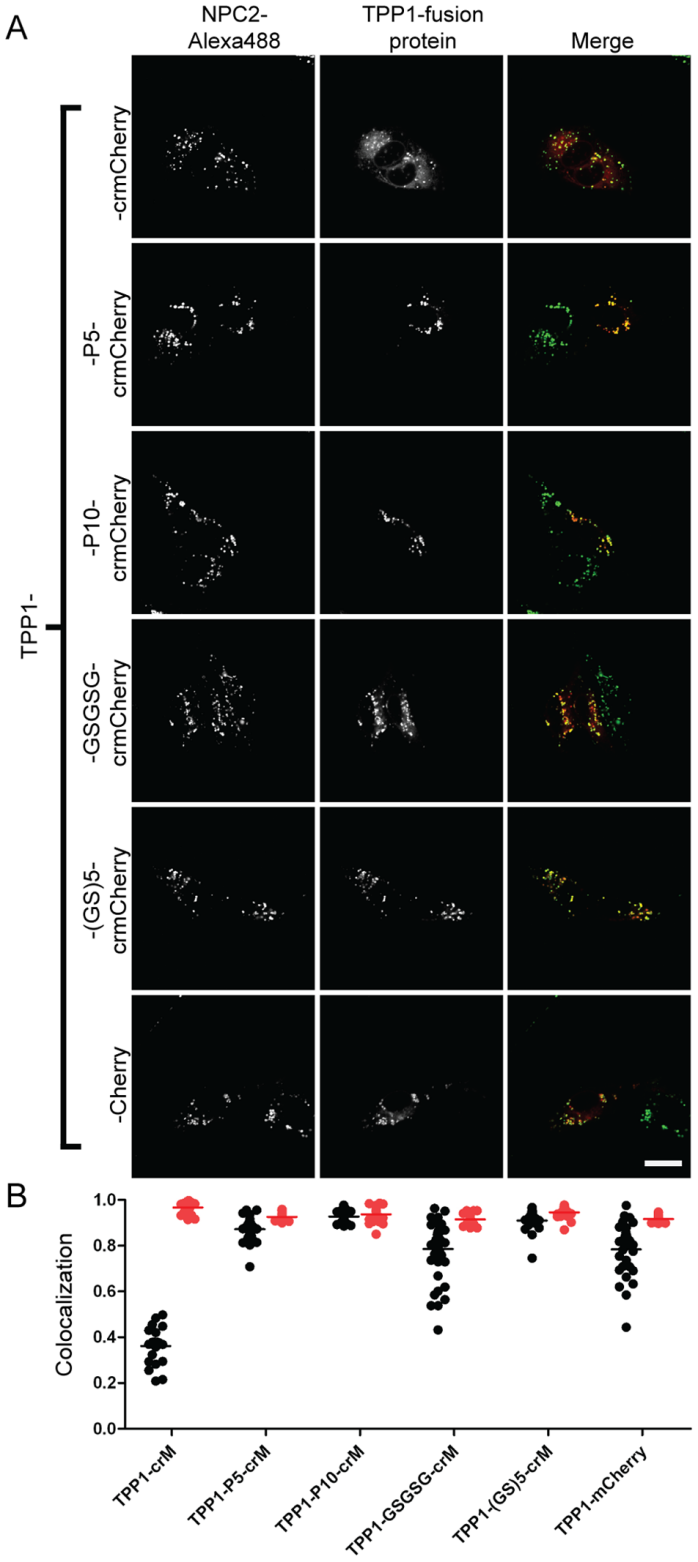
Linker sequences affect lysosomal targeting of TPP1 fusion proteins. A) U2OS cells were transiently transfected with indicated fusion constructs. Linker sequences are as described in Fig. 3 legend. Endocytosed Alexa488-NPC2 is used as a lysosomal standard. The scale bar (white) in the bottom right corner represents 20 µm. B) Quantitative co-localization analysis. Mander’s colocalization coefficients (tM) were plotted for individual fields of cells. Each dot represents analysis of a single field containing 1–4 transfected cells. Black dots (tM1) represent Mander’s coefficients for the fraction of pixels containing red signal from crmCherry/mCherry that also contained signal from green-NPC2, while red dots (tM2) represent Mander’s coefficients for the fraction of lysosomes (marked by green-NPC2) that also contain red fluorescent protein chimera. The number of cells analyzed for each fusion construct are as follows: TPP1-crM (33), TPP1-P5-crM (37), TPP1-P10-crM (43), TPP1-GSGSG-crM (43), TPP1-(GS)5-crM (34), TPP1-mCherry (33).

To investigate if crmCherry could be a general tool for studying lysosomal proteins, we extended our analysis to the lysosomal protease TPP1, creating a similar C-terminal tag fusion as described for NPC2. Since both imaging and western blot analysis yielded similar results for NPC2 fusion proteins tested in CHO, U2OS and MEF cells (Fig. 1B, Figs. 2–4, Figs. S1–S2), U2OS cells were used for imaging due to their superior morphology when cultured at low densities and CHO cells were used for biochemical analysis due to their more robust levels of expression. Imaging revealed that a large proportion of the TPP1-crmCherry was localized outside of the lysosome (mean tM1 = 0.36) (Fig. 5). We hypothesized that the direct C-terminal fusion of crmCherry to TPP1 could interfere with trafficking and thus we engineered five additional constructs with linkers between TPP1 and crmCherry: P5, P10, GSGSG, (GS)5 and the 11-amino terminal residues of mCherry. We observed that the addition of all linkers tested improved lysosomal localization, with TPP1-P10-crmCherry having a mean tM1 of 0.9 (Fig. 5).

### Effects of mCherry Tagging on TPP1 Secretion and M6P Processing

**Figure 6:**
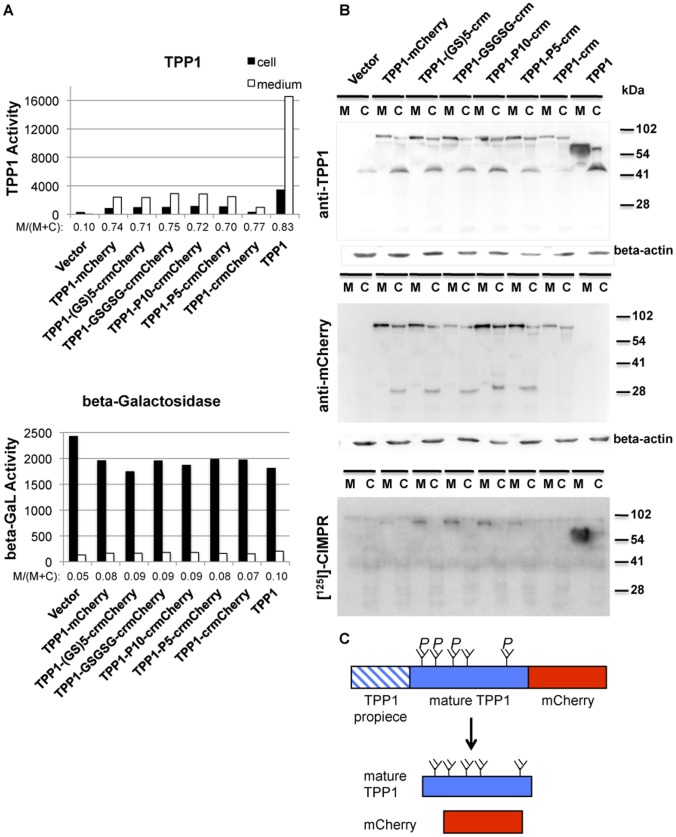
Expression of TPP1 fusion proteins. CHO cells were transiently transfected as indicated. Media was collected at 24) TPP1 and β-galactosidase activity measured in media and cell lysates. Data represents total activity in media (24 and 48-hour collection points) and cells. B) Blot analysis of cell lysates and 48-hour collection point for media samples. Note that ∼5 times greater proportional equivalence of cell lysate was loaded compared to media samples. C) Illustration of processing of TPP1 fusion proteins in the lysosome observed in Panel B. Glycans are labeled with or without P to indicate removal of the M6P modification. Note that this is for heuristic purposes only and does note imply stoichiometry or sites of phosphorylation.

Given that direct fusion of crmCherry to TPP1 affects targeting to the lysosome, it was important to determine whether the tag affected protein secretion. We therefore analyzed TPP1 enzyme activity in cell extracts and media of transiently transfected CHO cells (Fig. 6A, Top Panel). Note that media was incubated at a low pH prior to TPP1 assay to activate the proenzyme [19]. The lysosomal enzyme β-galactosidase was also measured as a control (Fig. 6A, Bottom Panel). In the vector control, the cell lysate contains more enzyme activity, and therefore more endogenous enzyme, than the medium. In contrast, transfected untagged rhTPP1 and the TPP1 fusion proteins have significantly greater secretion, with 70–80% of the total activity found in the media.

If mCherry fusion affected TPP1 enzymatic activity, then this measurement might not reflect protein distribution. Thus, we also conducted western blot analysis using α-TPP1 and α-mCherry antibodies. In cells transfected with TPP1, the 66 kDa proTPP1 is largely secreted into the medium. The proenzyme is largely processed to the 46 kDa, mature TPP1 within the cells (Fig. 6B Top Panel). For the fusion proteins, a ∼97 kDa band is present in the media. Based on its size and detection by both α-TPP1 and α-mCherry antibodies, this likely represents the proTPP1-mCherry chimera, which was confirmed by incubating the media at low pH to induce TPP1 autoactivation (Fig. S3). Interestingly, in the cells, there is little evidence of the expected intact mature ∼76 kDa TPP1-mCherry chimeras, while the 46 kDa mature TPP1 and cleaved mCherry are apparent (Fig. 6B Top and Middle Panel). In contrast, the majority of NPC2 is retained in cells, with only a minority secreted (Fig. S4).

The reason why the transfected TPP1 and associated fluorescent chimeras are largely secreted rather than being targeted to the lysosome is unclear. However, we have previously found that the degree of TPP1 secretion increases with increasing levels of TPP1 expression in CHO cells, even though the secreted proenzyme contains the M6P modification and can be readily endocytosed by other cells [20]. Multiple other lysosomal proteins are also largely secreted when overexpressed [21].

TPP1 differs markedly from NPC2 in that, in the cell at least, crmCherry is released from the fusion regardless of the linker sequence. One possibility is that the fusion protein interferes with oligomerization of mature TPP1 following targeting to the lysosome and conversion of the proenzyme to the proteolytically processed catalytically active form. The TPP1 proenzyme is a soluble monomer [20]. In contrast, the mature TPP1 forms oligomers [22], [23] that may be important for stability. Thus, at steady state, western blotting may detect only that portion of the chimera that has been cleaved to release the fluorescent tag.

To determine if tagging interferes with addition of the M6P modification to TPP1, we probed cell lysate and medium samples with a radioiodinated soluble form of the cation-independent M6P receptor (sCI-MPR). Relatively low amounts of M6P glycoproteins are detected in cells as the modification is quickly removed in the lysosome by acid phosphatase 5 (ACP5) in most cell types [24]. For TPP1, the 66 kDa proform contains M6P as do the ∼97 kDa proforms of the TPP1 fusion proteins with the exception of TPP1-crmCherry (Fig. 6B, Bottom Panel).

**Figure 7:**
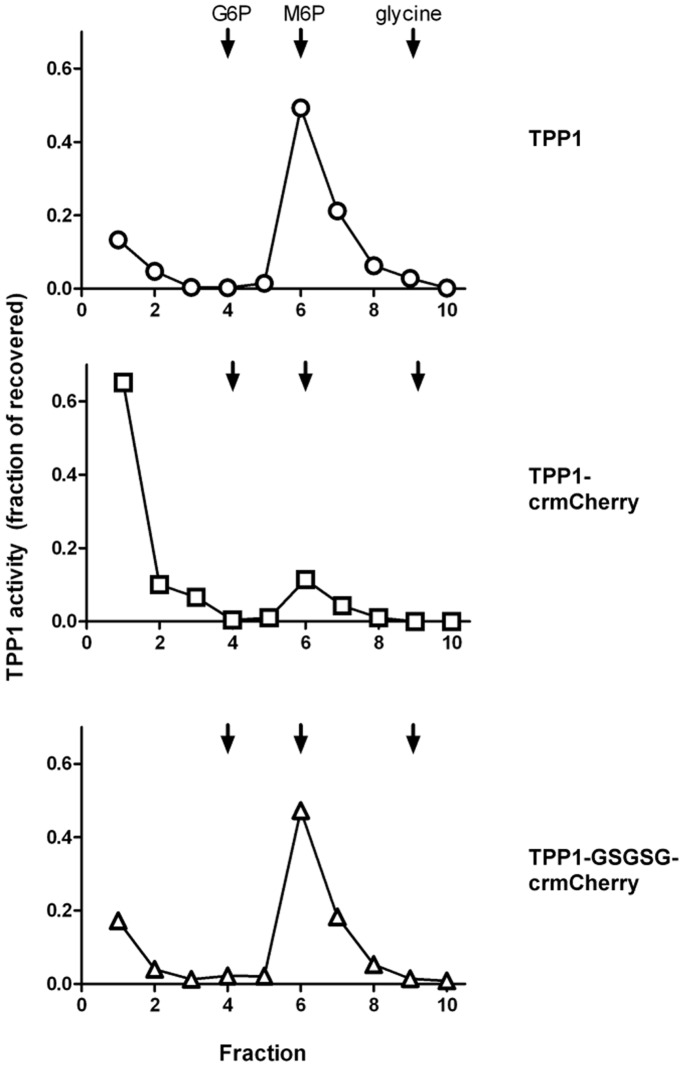
M6P content of TPP1 fusion proteins. CHO cells were transiently transfected with the indicated constructs. The media samples collected at the two time points as in Fig. 6 were pooled and used for MPR-affinity chromatography. Fractions (1, flow through; 2 and 3, washes; 4 and 5, G6P/mannose mock elution; 6–8, specific elution with M6P; 9 and 10, glycine elution) were analyzed for TPP1 activity after preactivation.

The portion of secreted TPP1, TPP1-crmCherry, and TPP1-GSGSG-crmCherry that was recognized by the M6P receptor was determined by affinity chromatography on immobilized sCI-MPR (Fig. 7). Most of the TPP1 and the TPP1-GSGSG-crmCherry chimera bound to the sCI-MPR column and was specifically eluted with M6P. In contrast, less than 20% of the TPP1-crmCherry fusion protein specifically bound to the column. This strongly suggests that direct fusion of crmCherry to TPP1 either blocks generation of the M6P modification and/or binding of M6P receptors to the phosphorylated N-linked glycans. This may arise due to steric effects or to structural changes induced by the proximity of mCherry. Regardless, addition of linkers relieves this hindrance. This would explain the significant degree of non-lysosomal localization of TPP1-crmCherry (Fig. 5).

### Structural Modeling

Our data show that the degree to which TPP1-mCherry chimeras are targeted to the lysosome is influenced by the length and sequence of the linker. In contrast, NPC2-crmCherry fusions effectively reach the lysosome regardless of the type of linker used. One plausible explanation for this difference lies in the respective locations of oligosaccharides in the two proteins relative to the fluorescent protein fusion site. Mammalian NPC2 proteins have one conserved N-linked glycosylation site on Asn39 that bears the M6P targeting modification [17]. The crystal structure of TPP1 revels that four glycosylation sites are utilized [19], [25], and mutagenesis studies indicate that the site at Asn286 is particularly critical for folding and targeting [26].

**Figure 8:**
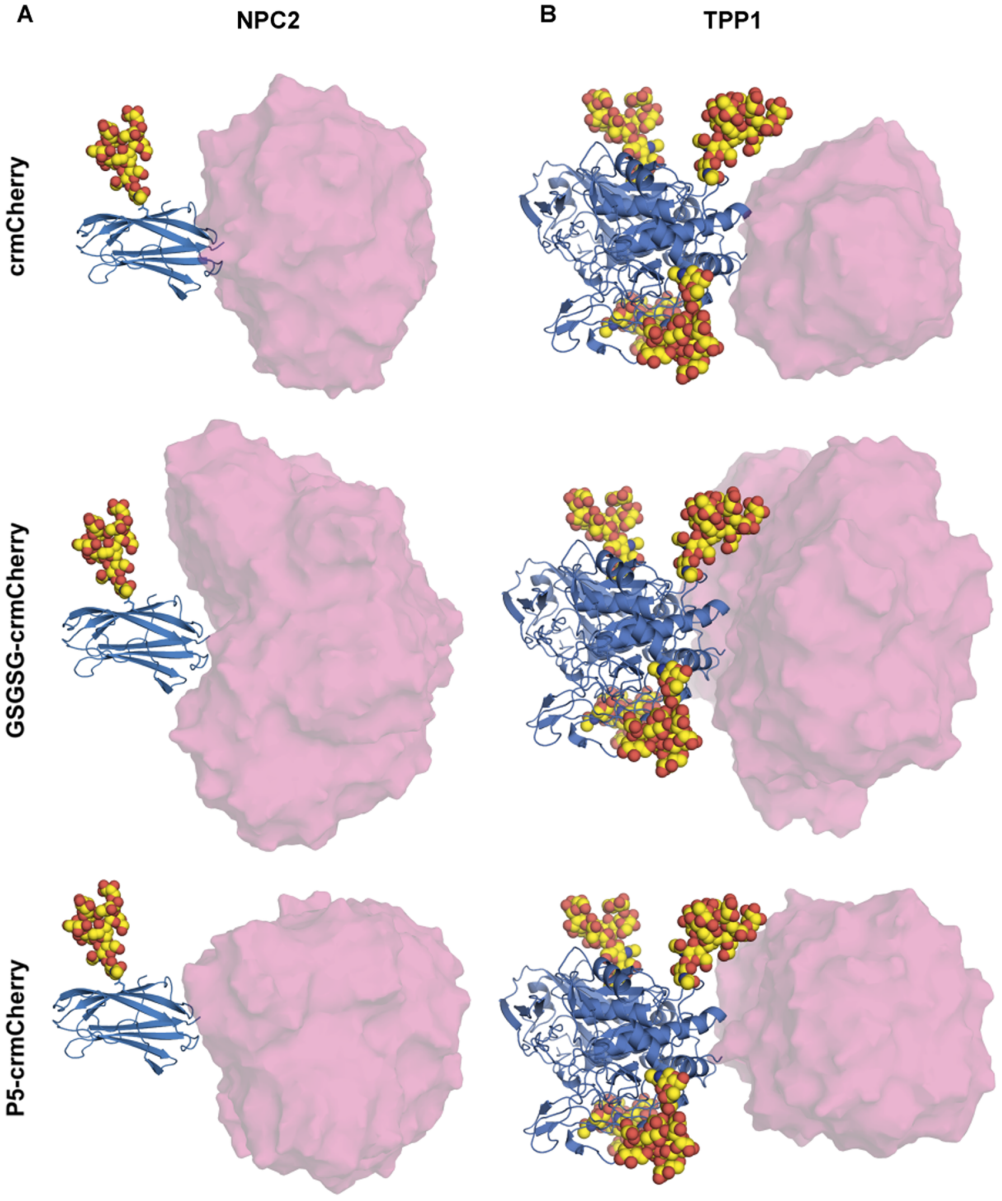
Modeling of fusion protein constructs. Models of NPC2 (A) and TPP1 (B) directly fused to crmCherry, or separated by Pro or Ser and Gly linkers, were generated by merging protein structures with the low energy configurations of high mannose oligosaccharides placed at the common coordinates of GlcNAc groups. For TPP1, the oligosaccharides linked to Asn286 and Asn313 are closest to mCherry and are oriented pointing up and down, respectively. Envelopes of mCherry represent 50 randomly chosen structures from the allowable modeled conformations.

Structural modeling of the various NPC2-crmCherry chimeras indicates that the attached fluorescent protein is spatially distinct from and unable to interact with the high-mannose oligosaccharide located on Asn39 (Fig 8a). In contrast, oligosaccharides bound to Asn286 and Asn313 of TPP1 are adjacent to the crmCherry fusion site (Fig 8b). As a result, a significant fraction of linker conformations result in structural clashes between the oligosaccharide and crmCherry. Given this close proximity, many of the linker conformations result in crmCherry occluding one of the two glycosylation sites. This may adversely impact trafficking by blocking M6P receptor binding, or disrupting appropriate glycosylation/modification at these sites. The extent of correct lysosomal localization of TPP1-crmCherry fusion correlates with the degree to which steric clashes between crmCherry and oligosaccharide constrain the flexibility of the linker. The P5 linker is intrinsically rigid due to constraints of proline on peptide chain conformation. Approximately one-fifth of allowable TPP1-P5-crmCherry conformations are unhindered, with no clashes between crmCherry and either TPP1 or attached oligosaccharides (Table 1). The greater intrinsic flexibility of the GSGSG linker allows for more clashes between proteins and oligosaccharides; one tenth of allowed conformations are unhindered. Only 2% of direct TPP1-crmCherry fusion conformations did not exhibit intramolecular steric clashes. Thus, the extent of correct trafficking of TPP1 fusions to the lysosome can be rationalized based on two factors: the entropic cost of restricting linker conformations (P5< GSGSG<direct fusion), and the close proximity of the fluorescent protein to bound oligosaccharides. This is also consistent with effective localization of chimeras with P10 and (GS)5 linkers, although these were not computationally modeled.

**Table 1:** Allowable number of linker conformations in the absence and presence of fusion proteins*.

		No tag	+ crmCherry
	Direct fusion**	8279	161 (2%)
**TPP1**	PPPPP	8973	1853 (21%)
	GSGSG	23169	2165 (9%)
	Direct fusion**	7948	847 (11%)
**NPC2**	PPPPP	8973	5741 (64%)
	GSGSG	23169	12522 (54%)

*Note that while the absolute numbers of conformations are dependent on the parameters used for modeling, the relative magnitudes can be directly compared. Relative magnitudes (in parentheses) are calculated by dividing the value of Protein+crmCherry by the value of Protein with linker alone (no tag).

**For constructs of direct fusion, the last 2 amino acid residues at the C-terminus of TPP1 or NPC2 and the first 3 residues at the N-terminus of crmCherry were used for the analysis.

### Use of Fusion Proteins to Monitor Lysosomal Localization

**Figure 9:**
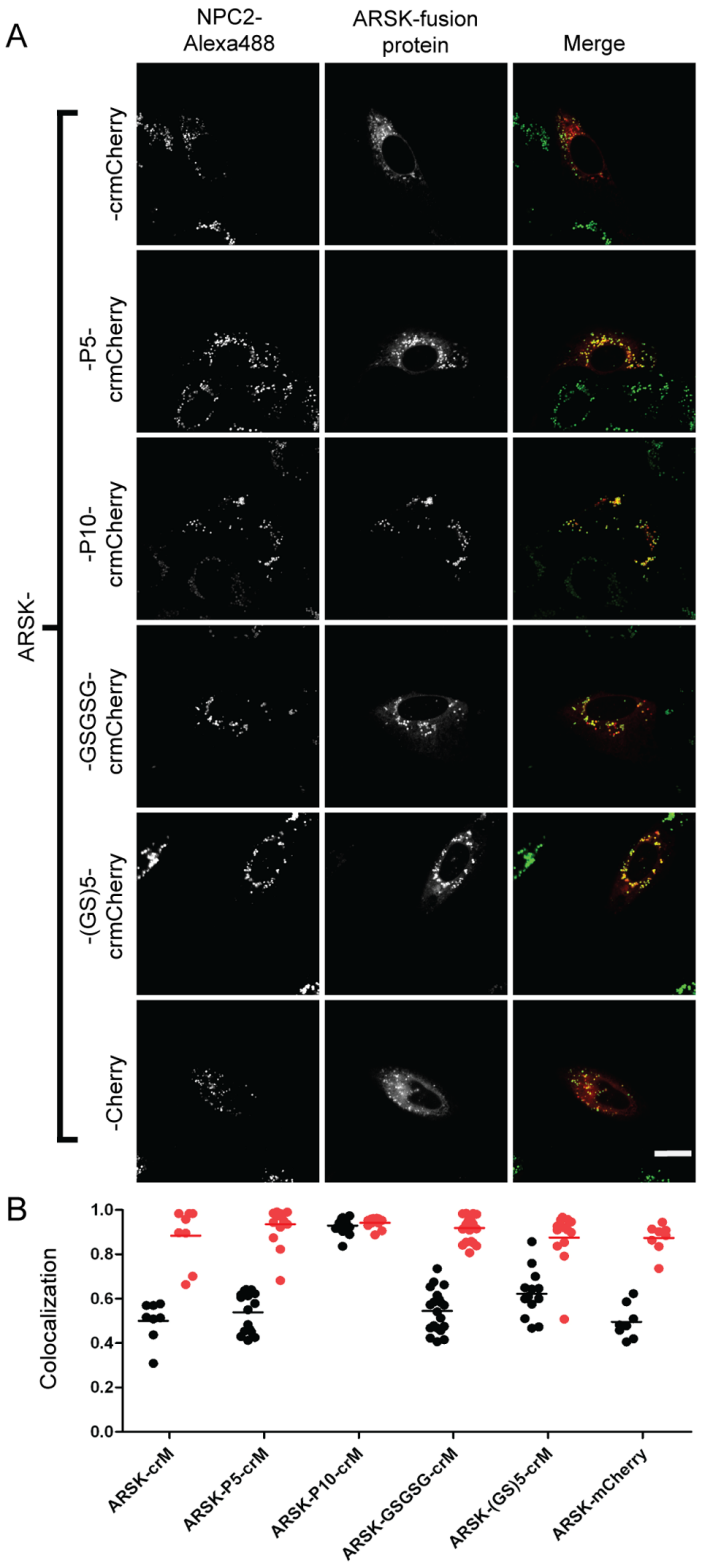
Intracellular localization of ARSK fusion proteins. A) U2OS cells were transiently transfected with indicated fusion constructs. Linker sequences are as described in Fig. 3 legend. Endocytosed Alexa488-NPC2 is used as a lysosomal standard. The scale bar (white) in the bottom right corner represents 20 µm. B) Quantitative co-localization analysis. Mander’s colocalization coefficients (tM) were plotted for individual fields of cells. Each dot represents analysis of a single field containing 1–4 transfected cells. Black dots (tM1) represent Mander’s coefficients for the fraction of pixels containing red signal from crmCherry/mCherry that also contained signal from green-NPC2, while red dots (tM2) represent Mander’s coefficients for the fraction of lysosomes (marked by green-NPC2) that also contain red fluorescent protein chimera. The number of cells analyzed for each fusion construct are as follows: ARSK-crM (10), ARSK-P5-crM (16), ARSK-P10-crM (12), ARSK-GSGSG-crM (20), ARSK-(GS)5-crM (13), ARSK-mCherry (11).

**Figure 10:**
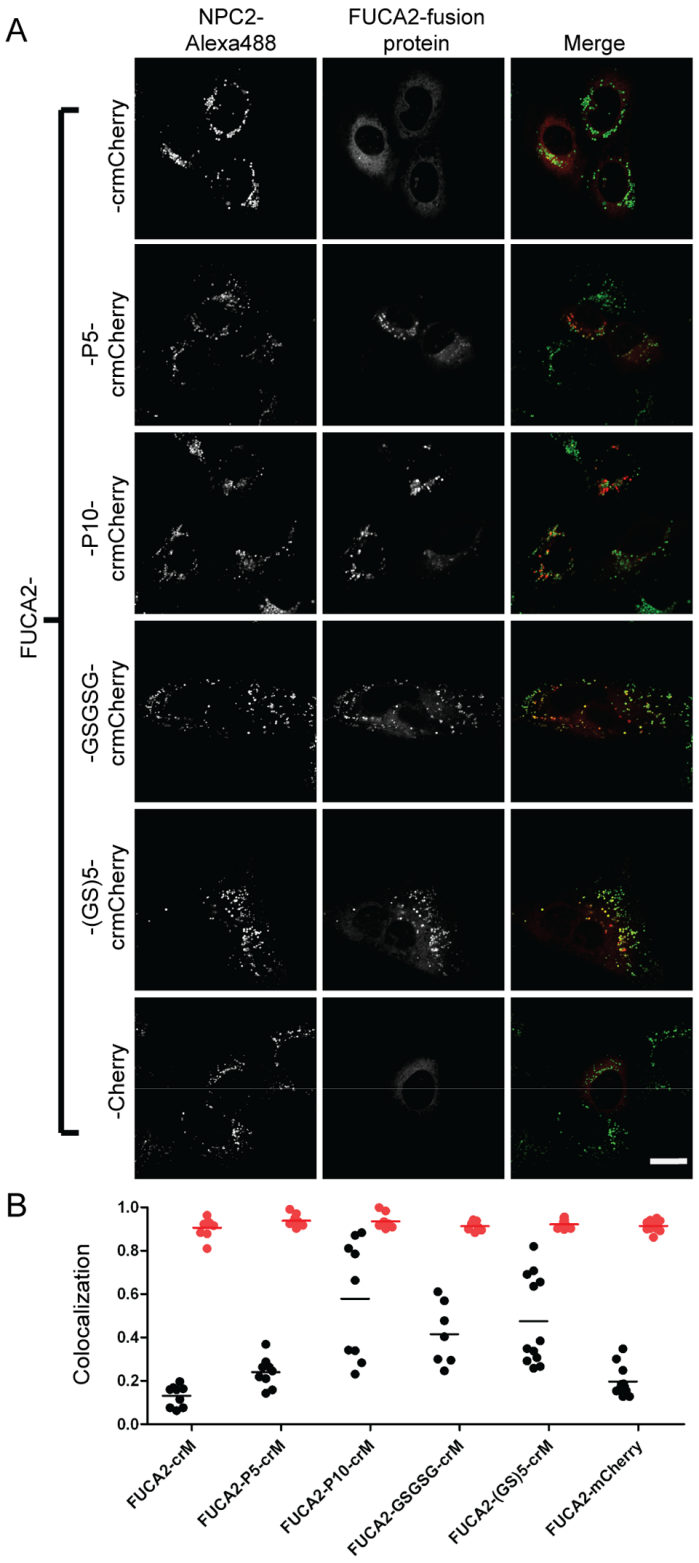
Intracellular localization of FUCA2 fusion proteins. A) U2OS cells were transiently transfected with indicated fusion constructs. Linker sequences are as described in Fig. 3 legend. Endocytosed Alexa488-NPC2 is used as a lysosomal standard. The scale bar (white) in the bottom right corner represents 20 µm. B) Quantitative co-localization analysis. Mander’s colocalization coefficients (tM) were plotted for individual fields of cells. Each dot represents analysis of a single field containing 1–4 transfected cells. Black dots (tM1) represent Mander’s coefficients for the fraction of pixels containing red signal from crmCherry/mCherry that also contained signal from green-NPC2, while red dots (tM2) represent Mander’s coefficients for the fraction of lysosomes (marked by green-NPC2) that also contain red fluorescent protein chimera. The number of cells analyzed for each fusion construct are as follows: FUCA2-crM (13), FUCA2-P5-crM (15), FUCA2-P10-crM (12), FUCA2-GSGSG-crM (13), FUCA2-(GS)5-crM (20), FUCA2-mCherry (16).

**Figure 11:**
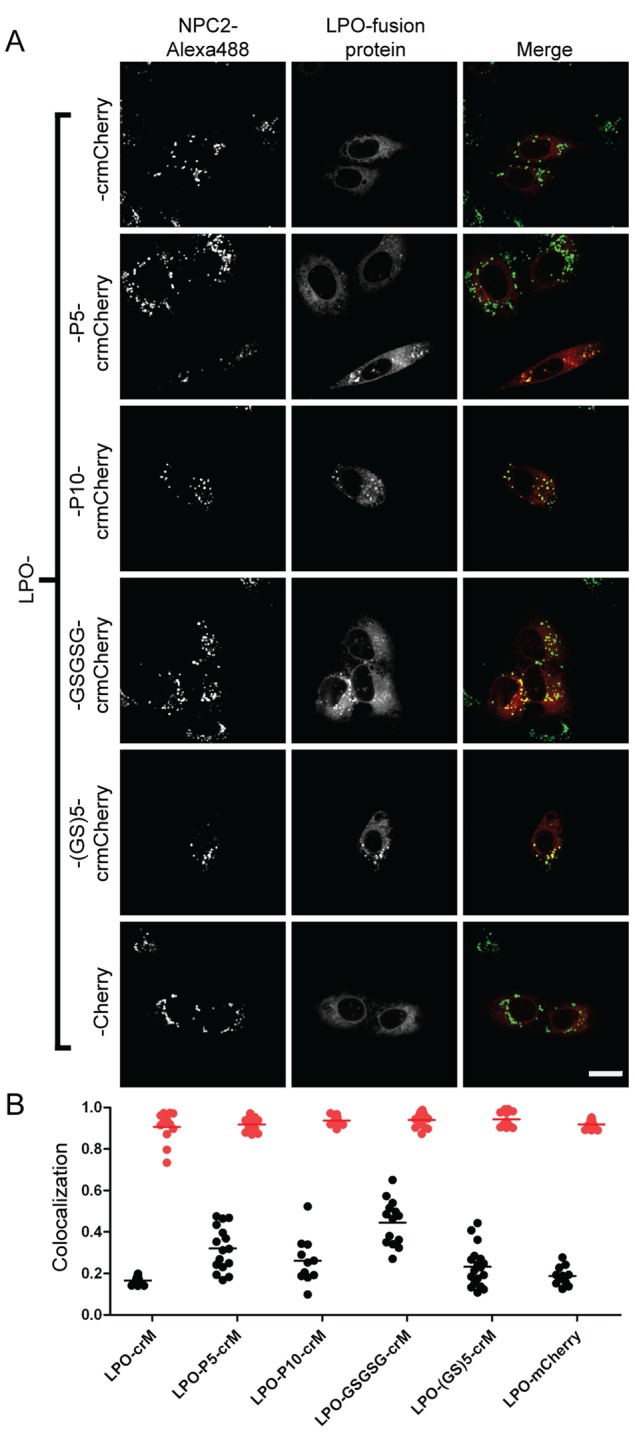
Intracellular localization of LPO fusion proteins. A) U2OS cells were transiently transfected with indicated fusion constructs. Linker sequences are as described in Fig. 3 legend. Endocytosed Alexa488-NPC2 is used as a lysosomal standard. The scale bar (white) in the bottom right corner represents 20 µm. B) Quantitative co-localization analysis. Mander’s colocalization coefficients (tM) were plotted for individual fields of cells. Each dot represents analysis of a single field containing 1–4 transfected cells. Black dots (tM1) represent Mander’s coefficients for the fraction of pixels containing red signal from crmCherry/mCherry that also contained signal from green-NPC2, while red dots (tM2) represent Mander’s coefficients for the fraction of lysosomes (marked by green-NPC2) that also contain red fluorescent protein chimera. The number of cells analyzed for each fusion construct are as follows: LPO-crM (30), LPO-P5-crM (22), LPO-P10-crM (12), LPO-GSGSG-crM (25), LPO-(GS)5-crM (22), LPO-mCherry (16).

In previous studies, we used fluorescent protein chimeras to investigate the subcellular location of candidate lysosomal proteins identified from proteomic analysis of affinity purified M6P glycoproteins [27]. Varying degrees of lysosomal location were found for candidates fused with crmCherry. Given our findings with TPP1, we extended this analysis with some of these and other candidates using different linkers, focusing on several with relatively low lysosomal localization when fused directly to crmCherry. We analyzed fusions of arylsulfatase K (ARSK), alpha-L-2 fucosidase (FUCA2), or lactoperoxidase (LPO) with crmCherry. Linker addition resulted in increasing lysosomal localization for all these proteins albeit to different degrees. For ARSK and FUCA2, the fusion protein containing the P10 linker exhibited the most colocalization with the lysosomal marker (Figs. 9 and 10), indicating that these are resident lysosomal proteins. For ARSK, this conclusion is supported by a recent study demonstrating a lysosomal localization for ARSK using immunohistochemistry [28]. For LPO, the GSGSG linker resulted in the greatest lysosomal localization (Fig. 11). Note that in all cases, the direct mCherry fusion protein (containing the 11 N-terminal residues of mCherry) and the direct crmCherry had similar, mostly non-lysosomal, distributions. Unlike NPC2, based on α-mCherry immunoblotting of all constructs expressed in CHO cells, we did not see significant degradation or cleavage in ARSK, FUCA2 or LPO fusion proteins with crmCherry (data not shown). While these results indicate that linker addition can enhance lysosomal localization of fusion proteins, the effect of a given linker is protein-dependent.

**Figure 12:**
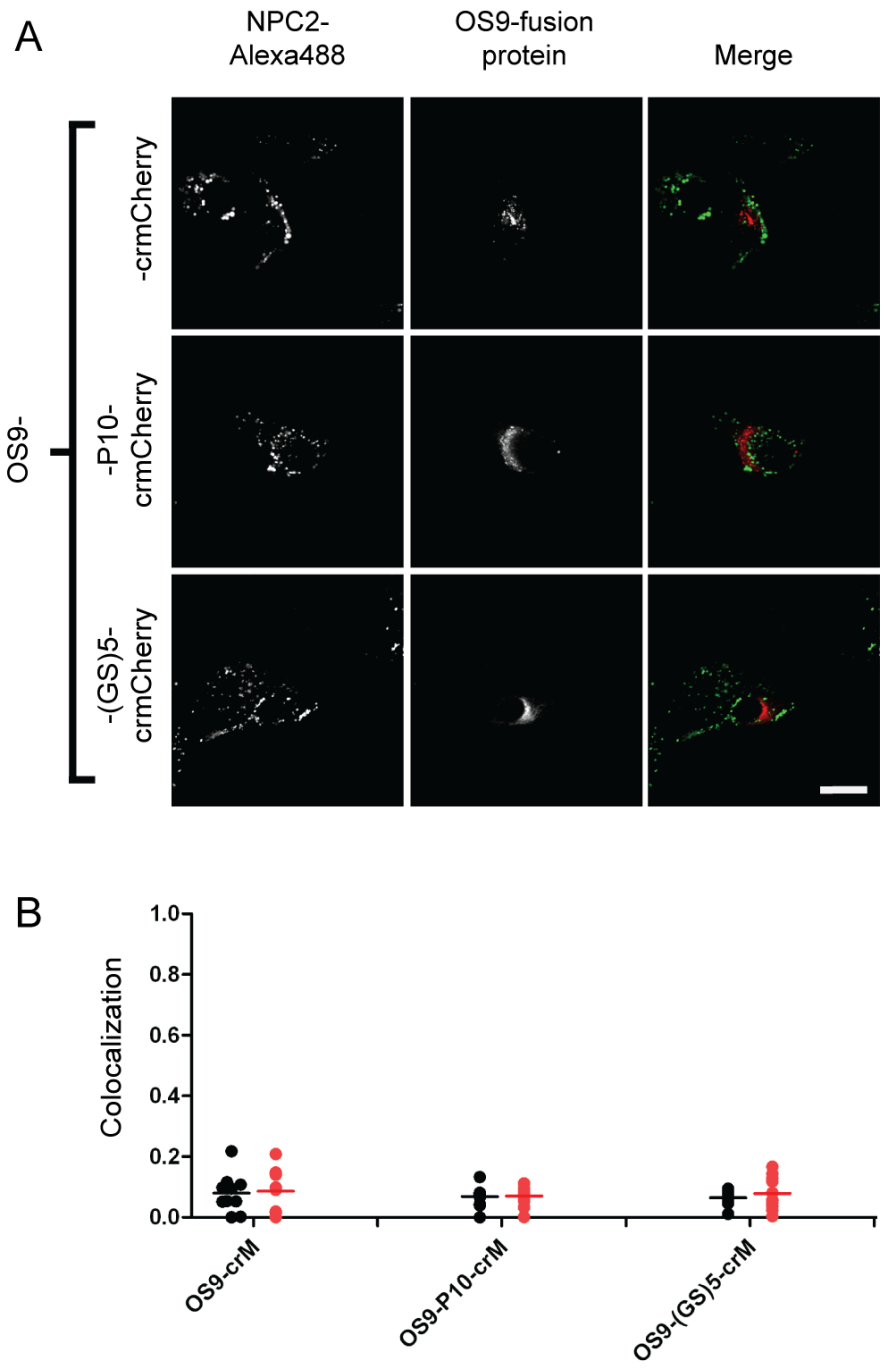
Intracellular localization of OS9 fusion proteins. A) U2OS cells were transiently transfected with indicated fusion constructs. Linker sequences are as described in Fig. 3 legend. Endocytosed Alexa488-NPC2 is used as a lysosomal standard. The scale bar (white) in the bottom right corner represents 20 µm. B) Quantitative co-localization analysis. Mander’s colocalization coefficients (tM) were plotted for individual fields of cells. Each dot represents analysis of a single field containing 1–4 transfected cells. Black dots (tM1) represent Mander’s coefficients for the fraction of pixels containing red signal from crmCherry/mCherry that also contained signal from green-NPC2, while red dots (tM2) represent Mander’s coefficients for the fraction of lysosomes (marked by green-NPC2) that also contain red fluorescent protein chimera. The number of cells analyzed for each fusion construct are as follows: OS9-crM (10), OS9-P10-crM (12), OS9-(GS)5-crM (12).

To address if the fusion proteins and/or linkers could cause aberrant targeting of non-lysosomal proteins to the lysosome, we engineered crmCherry to the C-terminus of osteosarcoma amplified 9 (OS9), an endoplasmic reticulum (ER) resident protein [29], [30] and chemokine C-C motif ligand 2 (CCL2), which is primarily secreted [31], [32]. OS9 chimeras with P10, (GS)5, or no linker did not colocalize with the lysosomal marker but exhibited a pattern typical of ER (Fig. 12). Similar fusions with CCL2 were essentially undetectable within the cell (Fig. S5). Analysis using α-mCherry demonstrated robust expression with secretion into the media (Fig. S5). These results indicate that the crmCherry or linker variants do not promote artifactual lysosomal targeting.

### Conclusions

Interpretation of studies using fusion proteins can be complicated when the intact chimeras have different fates from the endogenous proteins of interest. This is particularly relevant for lysosomal proteins, which have evolved to withstand the environment of this digestive organelle. mCherry has previously been used as a probe which retains its fluorescence in the lysosome. However, in this study we demonstrate that there are multiple potential problems in the use of mCherry to study lysosome proteins, and that in some cases these can be minimized through judicious design and validation of constructs.

We have found that the N-terminal region of full-length mCherry is susceptible to cleavage within the lysosome. This is not necessarily a problem if the experimental aim is to determine if a fusion protein reaches the lysosome. For instance, we find that fusions between mCherry and either NPC2 or TPP1 undergo significant cleavage to release mCherry, but in both cases, the majority of the fluorescence colocalizes with the lysosomal marker in morphological studies. However, if the goal is to study more complex trafficking events in which a protein of interest may visit the lysosome prior to being routed to other compartments, then the cleavage clearly is a cause of concern. Here, the destination of the liberated fluorescent tag and the tag-free protein may diverge after exposure to the lysosome. For NPC2, and potentially other proteins, use of the crmCherry variant preserves the integrity of the chimera and thus the distribution and dynamics determined by fluorescence imaging may more faithfully reflect that of the endogenous protein.

We also find that choice of linkers can have significant impact on the degree of cleavage as well as lysosomal targeting. For NPC2, elimination of linker or use of a proline linker helps to reduce release of crmCherry without affecting lysosomal targeting. For TPP1, the linker-free crmCherry fusion protein was relatively poorly targeted to the lysosome, while constructs with flexible or rigid linkers were efficiently targeted. For the candidate lysosomal proteins ARSK and FUCA2 identified in proteomic analyses of purified M6P glycoproteins, lysosomal targeting of the fusion proteins was significantly increased when crmCherry was attached via a ten proline linker. Linker design also had an effect on targeting of another candidate lysosomal protein, LPO, where the GSGSG-crmCherry fusion had the greatest degree of lysosomal residence. It is worth noting that, in addition to lysosomal localization, a considerable portion of LPO was located outside of the lysosome regardless of construct design, consistent with a multimodal distribution (e.g., secretory and lysosomal).

For TPP1 and NPC2, we provide a rational structural basis for the differing effects of the linker sequences on lysosomal targeting and stability of the fusion constructs. While such an analysis at the onset might help direct in chimera design, we believe at this point that empirical testing of multiple variants in parallel remains the most efficient path towards obtaining suitable fluorescent protein constructs. Finally, while this study underscores the need for careful design and interpretation of studies designed to specifically investigate the lysosome, it is worth noting that the use of fluorescent proteins to investigate other cellular compartments and to answer other biological questions, may also be subject to similar experiment-based artifacts.

## Materials and Methods

### Construction of Plasmids

Fusion constructs were based upon pmCherry-N1 (Clontech, CA) and constructed using the In-Fusion cloning system (Clontech, CA) [33], [34]. The coding sequence of mouse NPC2 (EST clone # 2649564), human ARSK (EST clone # 40146360), LPO (EST clone # 40034365) and FUCA2 (EST clone # 4855296) were obtained from Thermo Fisher Scientific. The human TPP1 coding sequence was amplified from a cDNA library. Details of cloning of constructs are provided in Table S1. All primer sequences are listed in Table S2. For stable expression of the NPC2-crmCherry fusion protein, a puromycin selectable marker with upstream internal ribosome entry sequence was amplified from plasmid pWHE467 [35] and inserted into the XbaI site of pNPC2-crmCherry.

### Tissue Culture

Cells were cultured at 37°C in a humidified 5% CO_2_ incubator. MEFs were cultured in DMEM (cat# 11995 Invitrogen, CA) supplemented with 10% fetal bovine serum (cat# F0926, SIGMA, MO), 1x penicillin/streptomycin (cat# 15140, GIBCO, CA), 1x nonessential amino acids (cat# 11140, GIBCO, CA), and 55 µM 2-mercaptoethanol (cat# 21985023, GIBCO, CA). U2OS (catalog # HTB-96) and CHO (catalog # CCL-61) cell lines were from the ATCC (Manassas, VA) and were cultured in DMEM/F-12 (cat# D6421, Sigma, MO) supplemented with 10% fetal bovine serum, 1x penicillin/streptomycin and 1x L-Glutamine (cat# 25030, GIBCO, CA). Transfection was conducted via incubating the cells in Opti-MEM serum free medium (cat# 31985-070, Invitrogen, CA) supplemented with premixed Lipofectamine 2000 (cat# 11668-027, Invitrogen, CA) and plasmid DNA. After three hours incubation, cells were rinsed with PBS and cultured in complete medium.

Procedures related to mice were conducted in compliance with approved protocols from the Robert Wood Johnson Medical School Institutional Animal Care and Use Committee. *Npc2^−/−^* BALB/c MEFs were prepared from 13.5-day embryos and immortalized by stable transfection with pMSSVLT, which contains the SV40T/t coding sequence [36]–[38]. Immortalized *Npc2^−/−^* MEFs stably expressing mNPC2-crmCherry were selected by puromycin (cat# P8833, Sigma, MO) at 5 µg/ml for 2 weeks.

### Conjugation of rhNPC2 with Alexa Fluor 488

Purified rhNPC2 was reacted with Alexa Fluor 488 reagent N-hydroxy succinimide esters (NHS) for 1 hour at room temperature. The protein was separated from free dye and exchanged into PBS by gel filtration chromatography on PD-10 column (GE healthcare, UK). Absorbance readings indicated an average of 1.9 molecules Alexa Fluor 488 per molecule of NPC2.

### Confocal Imaging and Colocalization Analysis

Cells were grown to 90% confluence in 12-well tissue culture plates (BD Falcon, CA) before transfection with plasmid constructs using Lipofectamine 2000. After 3 hours incubation, media were removed and fresh growth media added. The next day, cells were plated on glass bottom image dishes (MatTek, MA) and cultured overnight in medium containing or lacking with 30 nM NPC2-Alexa488. Cells were washed 3 times with PBS and then cultured in phenol red free DMEM/F-12 (cat# D6434, Sigma, MO) supplemented with 10% fetal bovine serum, penicillin/streptomycin and Glutamax (GIBCO, CA). Four hours later, live images of cells in a 37°C/5% CO_2_ environment were obtained with a Zeiss LSM700 confocal microscope using a water immersion 63x objective.

Quantitative colocalization analysis was performed using the Colocalization Threshold plugin of NIH Image J [18] to calculate Mander’s colocalization coefficients. Only transfected cells with detectable green and red fluorescence were used for calculating coefficients, with at least ten cells analyzed per fusion protein (see Figure legends for details). Non-transfected cells present in some of the images as well as single-label controls (not shown) demonstrate the absence of fluorescent bleed-through. Colocalization analysis represents three independent experiments, including two individual wells of transfection for each of the three experiments.

### Filipin Staining

MEFs were cultured on 12-mm round coverslips (BD Biocoat, MA) for 2 days, rinsed three times with PBS, fixed with 3% paraformaldehyde in PBS for 30 min, washed three times with PBS, stained with 50 µg/ml filipin (Sigma, MO) in PBS at room temperature for 2 h, washed 3 times in PBS and coverslipped using Aqua Poly/Mount (Polysciences Inc. PA). Images were captured with a Nikon Eclipse 80 i microscope using a 40x objective.

### Blot Analysis

After transfection, cells in 6-well plates were cultured in 1 ml complete medium. Medium was collected after 24 h and replaced with fresh medium. After another 24 h, the medium was collected and adherent cells were washed three times with PBS, detached by trypsinization for 1 min at 37°C, suspended in 1 ml complete medium, and transferred to microcentrifuge tubes. Cells were pelleted and washed with PBS by three cycles of centrifugation and resuspension. Cell pellets were lysed in 200 µl ice-cold 0.15 M NaCl/0.1% Triton-100 containing 1 µg/ml pepstatin, 1 µg/ml leupeptin, 0.5 mM Pefabloc and 2.5 mM EDTA. Cleared lysates were prepared by centrifugation at 4°C 12,000 g for 5 min and stored at −80°C. Medium samples were centrifuged for 5 min at 12,000 g and supernatants stored at −80°C. Samples were reduced and denatured in LDS-sample buffer and fractionated by 10% Bis-Tris SDS-PAGE (Invitrogen, CA). Proteins were transferred to Immobilon-FL PVDF membranes (EMD Millipore, MA) using semidry blotting. Membranes were blocked with and probed in 5% bovine serum albumin (BSA) in 1×PBS/0.2% Tween-20. Primary rabbit antibodies were affinity-purified α-NPC2 (HL5873, 1∶4000), α-mCherry (CAT# 5993-100, BioVision, CA, 1∶200) and α-TPP1 antiserum (R72/5, 1∶1000) and were detected with Alexa 488-conjugated goat anti-rabbit IgG (CAT# A11008, Life technology, 1∶2000). Blots were washed 3×15 min with 1×PBS/0.2% Tween-20. Images were acquired using the FluorChem Q Imaging System (Alpha Innotech). As a loading control, blots were subsequently probed with mouse monoclonal anti-β-actin (CAT# 8H10D10, Abcam, MA. 1∶1000) and Alexa Fluor 680 conjugated donkey anti-mouse IgG (CAT# A10038, Invitrogen, 1∶500) and imaged as above. M6P glycoprotein blotting was conducted using ^[125I]^sCI-MPR as described [39] and analyzed using a Typhoon 9400 Variable Mode Imager (GE Healthcare).

### Enzyme Assays

Cell lysates and cleared media were harvested as described above. In media samples, proTPP1 was preactivated by incubating 2 h at pH 3.5 [19]. TPP1 was measured using Ala-Ala-Phe substrate 7-amido-4-methylcoumarin (Sigma, MO) in 0.15 mol/L NaCl/0.1% Triton X-100/pH 4.5 using an endpoint assay as described [40]. β-galactosidase activity was measured using substrate 4-methylumbelliferyl-β-D-galactopyranoside as described [41].

### MPR Affinity Chromatography

Immobilized sCI-MPR was used for affinity chromatography of M6P containing proteins, essentially as described [3]. Culture media was adjusted to contain 1×PBS, 0.2% Tween-20 and 5 mM β-glycerophosphate before administration to a 0.5 ml bed-volume column of immobilized sCI-MPR (2.5 mg/ml). The flow through was reloaded twice and column was washed with 2×1 ml of column buffer (1×PBS, 0.2% Tween-20 and 5 mM β-glycerophosphate). The column was sequentially eluted with 2×1 ml of 5 mM G6P/5 mM mannose in column buffer, 3×1 ml 10 mM M6P and then with 2×1 ml 100 M glycine pH 2.5.

### Structural Modeling

In order to determine the contribution of linker flexibility to allowable fusion protein conformations, models were constructed for NPC2 (starting with PDB structure ID 1NEP) and TPP1 (PDB ID 3EDY) fused to crmCherry (PDB ID 2H5Q) with the indicated five-residue linker. For the direct fusion, the two C-terminal residues of TPP1/NPC2 and three N-terminal residues of crmCherry were defined as the linker. Low energy configurations of high mannose oligosaccharides were constructed using GLYCAM Web [42] and merged with protein structures using the common coordinates of resolved GLcNAc groups on the NPC2 and TPP1 structures. Linker flexibility was modeled by sampling 10 low energy conformations per peptide bond (maximum of 10^5^ for the five residues) using the protein Computer Automated Design platform (protCAD) [43]. Low energy conformations were obtained from a set of high resolution X-ray diffraction structures. Steric clashes were specified based on AMBER 95 [44] definitions for atomic radii. Table 1 lists the number of conformations that did not exhibit steric clashes.

## Supporting Information

Figure S1 **Effect of linkers on NPC2 fusion protein stability.** *Npc2^−/−^* MEFs, U2OS, and CHO cells were transiently transfected with vector control or indicated fusion constructs. Linker sequences are as described in Fig. 2 and Fig. 3 legends. NPC2 and mCherry were detected in cell lysates by immunoblotting as described in Fig. 1 legend.
(TIF)

Figure S2 **Intracellular targeting of NPC2-crmCherry fusion protein is conserved among cell lines.** *Npc2^−/−^* MEF, U2OS and CHO cells were transiently transfected with a construct expressing NPC2-crmCherry. The fluorescent signal from the fusion proteins was compared with endocytosed Alexa488-NPC2 as a lysosomal marker. The scale bar (white) in the bottom right corner represents 20 µm.
(TIF)

Figure S3 **Autoactivation of TPP1 fusion proteins.** A) CHO cells were transiently transfected with mCherry or indicated TPP1 fusion constructs. Linker sequences are as described in legends to Figs. 1 and 3. Media samples were collected after 24 hours and were either activated (A) by incubation at low pH [19] prior to gel electrophoresis or analyzed without activation (A). Western blot was performed as Fig. 6B. B) Illustration of the auto-activation of TPP1 fusion proteins observed in Panel A.
(TIF)

Figure S4 **Effect of mCherry fusion on secretion of NPC2.** CHO cells were transiently transfected as indicated. Media was collected at 24 h, replaced with fresh media, and media and cell lysates collected after 48 hours. Blot analysis of cell lysates and 48-hour collection point for media samples. Note that ∼5 times greater proportional equivalence of cell lysate was loaded compared to media samples.
(TIF)

Figure S5 **Effect of mCherry fusion on secreted protein CCL2.** A) CHO cells were transiently transfected as indicated. Media was collected at 24 h, replaced with fresh media, and media and cell lysates collected after 48 hours. Blot analysis of cell lysates and 48-hour collection point for media samples. Note that ∼5 times greater proportional equivalence of cell lysate was loaded compared to media samples. The scale bar (white) in the bottom right corner represents 20 µm. B) U2OS cells were transiently transfected with indicated fusion constructs. Linker sequences are as described in Fig. 3 legend. Endocytosed Alexa488-NPC2 is used as a lysosomal standard.
(TIF)

Table S1 **Plasmid construction.**
(DOCX)

Table S2 **Sequence of oligonucleotide primers used in construct synthesis.**
(DOCX)
