# Supplementary figures and images for: Potential Pitfalls and Solutions for Use of Fluorescent Fusion Proteins to Study the Lysosome
Source: PLoS One. 2014 Feb 21;9(2):e88893. doi: 10.1371/journal.pone.0088893 (PMC3931630; doi:10.1371/journal.pone.0088893)

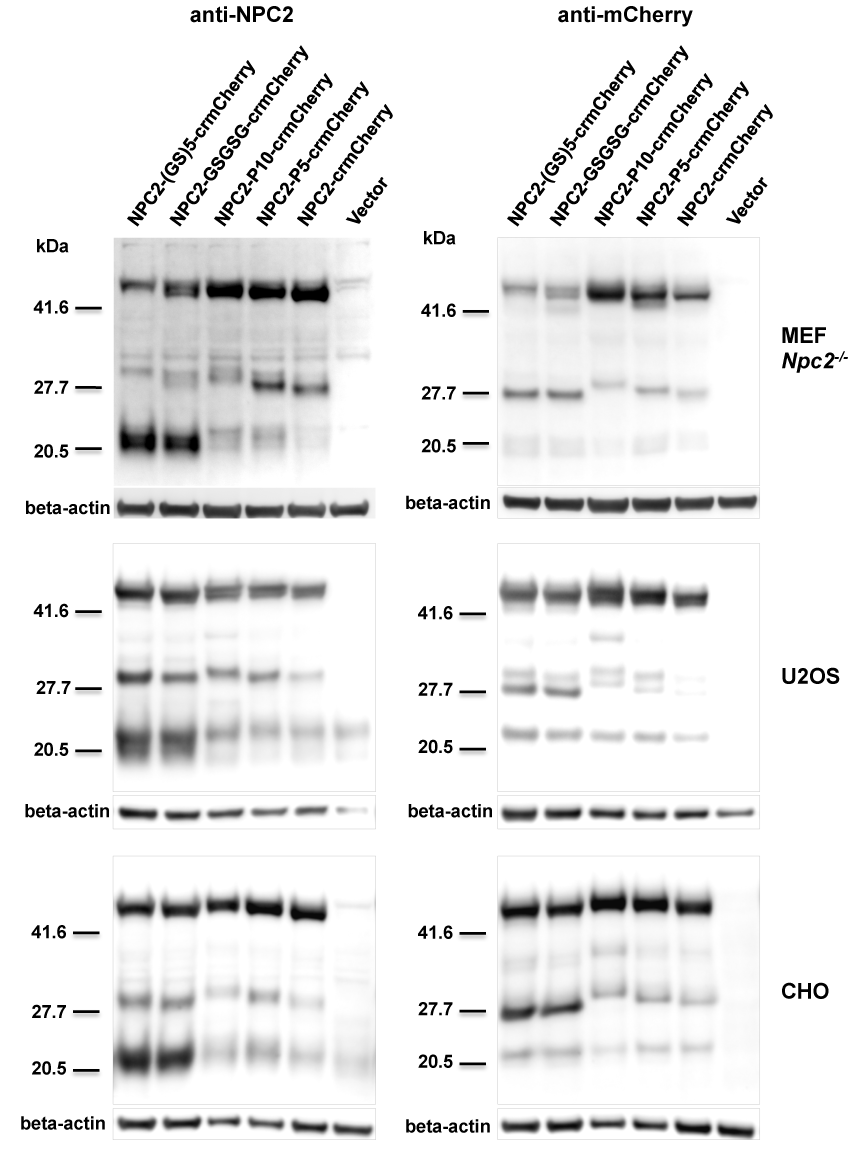

Supplement: Figure S1 — Effect of linkers on NPC2 fusion protein stability. Npc2−/− MEFs, U2OS, and CHO cells were transiently transfected with vector control or indicated fusion constructs. Linker sequences are as described in Fig. 2 and Fig. 3 legends. NPC2 and mCherry were detected in cell lysates by immunoblotting as described in Fig. 1 legend. (TIF) [file pone.0088893.s001.tif]

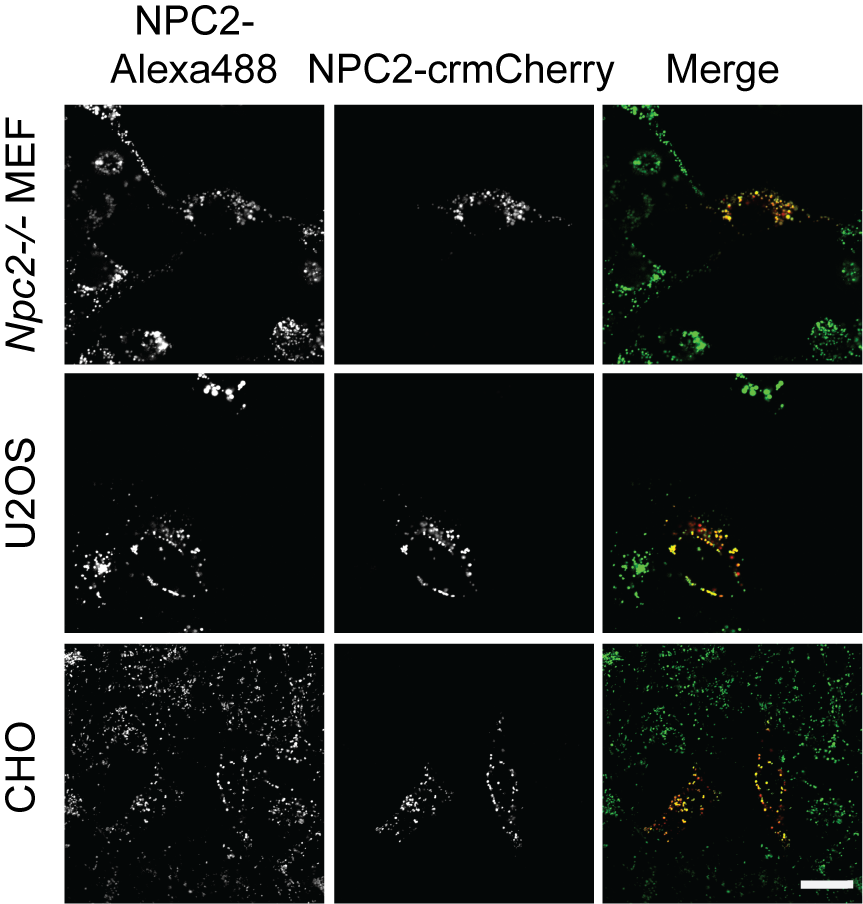

Supplement: Figure S2 — Intracellular targeting of NPC2-crmCherry fusion protein is conserved among cell lines. Npc2−/− MEF, U2OS and CHO cells were transiently transfected with a construct expressing NPC2-crmCherry. The fluorescent signal from the fusion proteins was compared with endocytosed Alexa488-NPC2 as a lysosomal marker. The scale bar (white) in the bottom right corner represents 20 µm. (TIF) [file pone.0088893.s002.tif]

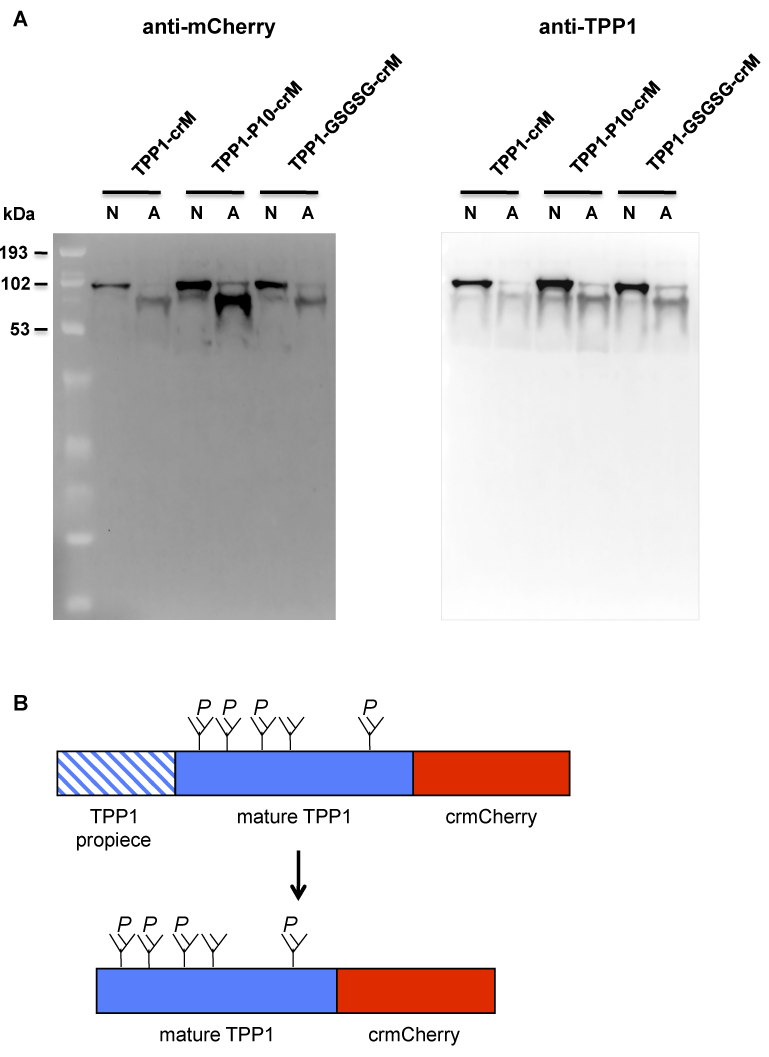

Supplement: Figure S3 — Autoactivation of TPP1 fusion proteins. A) CHO cells were transiently transfected with mCherry or indicated TPP1 fusion constructs. Linker sequences are as described in legends to Figs. 1 and 3. Media samples were collected after 24 hours and were either activated (A) by incubation at low pH [19] prior to gel electrophoresis or analyzed without activation (A). Western blot was performed as Fig. 6B. B) Illustration of the auto-activation of TPP1 fusion proteins observed in Panel A. (TIF) [file pone.0088893.s003.tif]

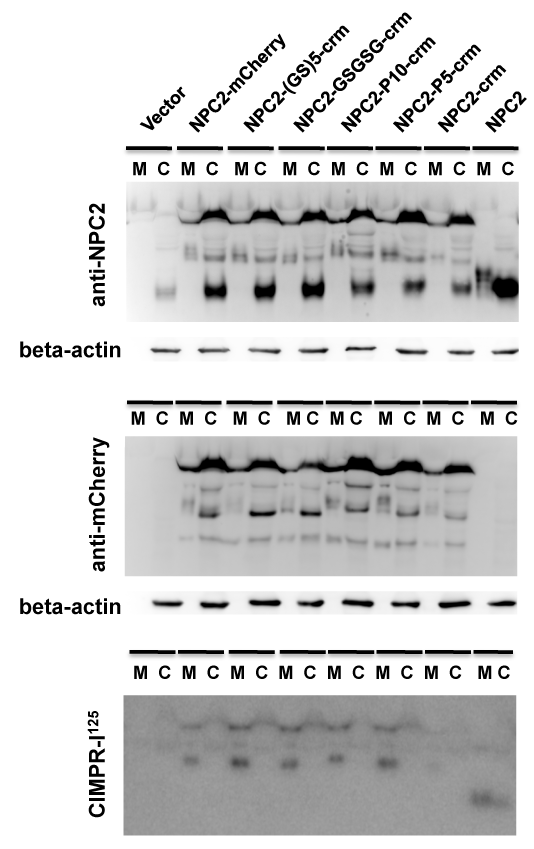

Supplement: Figure S4 — Effect of mCherry fusion on secretion of NPC2. CHO cells were transiently transfected as indicated. Media was collected at 24 h, replaced with fresh media, and media and cell lysates collected after 48 hours. Blot analysis of cell lysates and 48-hour collection point for media samples. Note that ∼5 times greater proportional equivalence of cell lysate was loaded compared to media samples. (TIF) [file pone.0088893.s004.tif]

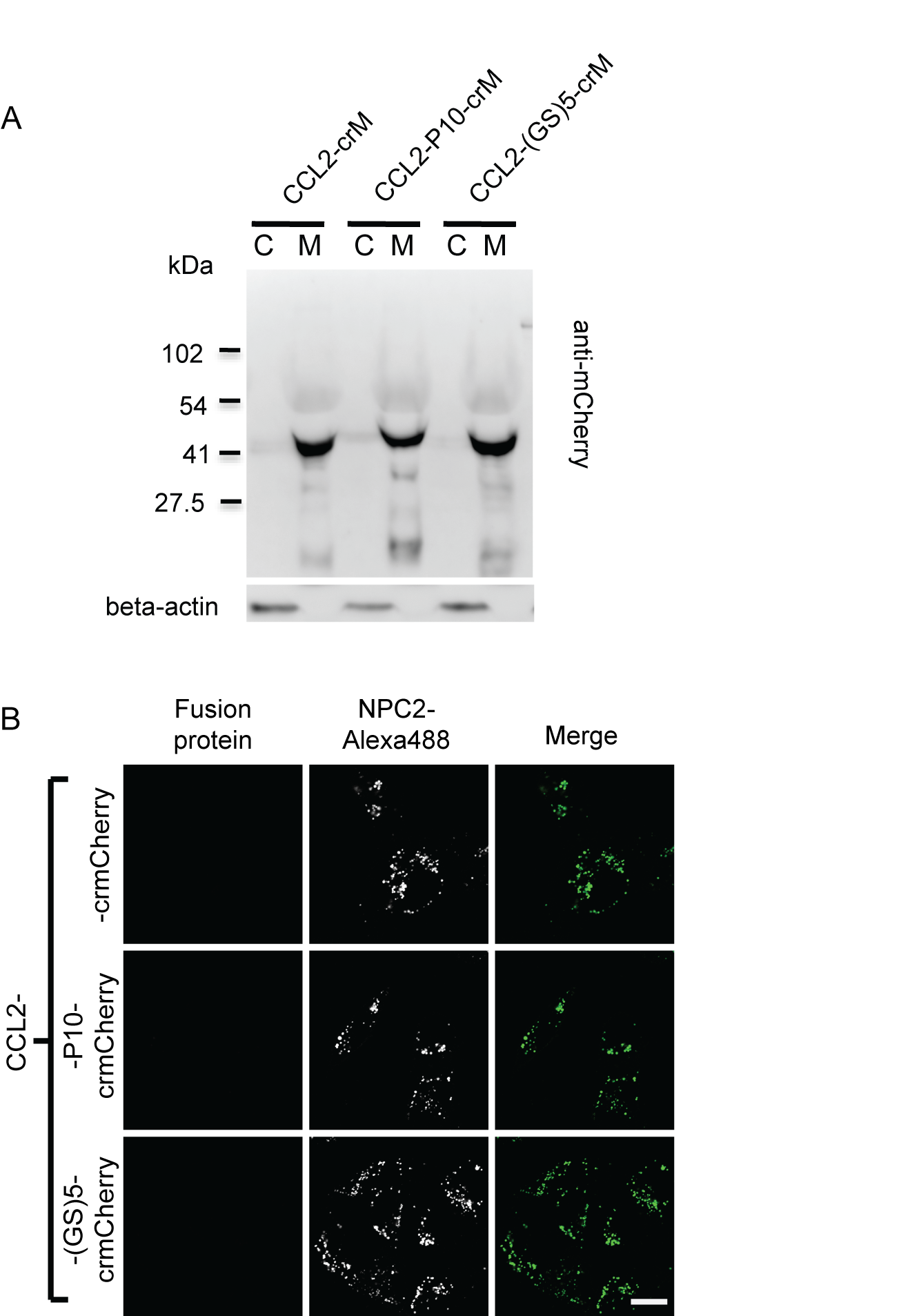

Supplement: Figure S5 — Effect of mCherry fusion on secreted protein CCL2. A) CHO cells were transiently transfected as indicated. Media was collected at 24 h, replaced with fresh media, and media and cell lysates collected after 48 hours. Blot analysis of cell lysates and 48-hour collection point for media samples. Note that ∼5 times greater proportional equivalence of cell lysate was loaded compared to media samples. The scale bar (white) in the bottom right corner represents 20 µm. B) U2OS cells were transiently transfected with indicated fusion constructs. Linker sequences are as described in Fig. 3 legend. Endocytosed Alexa488-NPC2 is used as a lysosomal standard. (TIF) [file pone.0088893.s005.tif]
