# Supplementary material for: Potential Pitfalls and Solutions for Use of Fluorescent Fusion Proteins to Study the Lysosome
Source: PLoS One. 2014 Feb 21;9(2):e88893. doi: 10.1371/journal.pone.0088893 (PMC3931630; doi:10.1371/journal.pone.0088893)
Supplement: Table S1 — Plasmid construction. (DOCX) [file pone.0088893.s006.docx]

**Table S1: Plasmid construction.**

|  | **Plasmid** | **Fragments used in plasmid construction** | | |
| --- | --- | --- | --- | --- |
|  |  | **Forward and reverse PCR primers, *template*** | | **Vector-containing fragment** |
| 1 | pNPC2-P10-crmCherry | F1-NPC2; R1-10P-NPC2-L; *pNPC2-crmCherry* | F2-10P-NPC2-L; R2-mCherry; *pNPC2-crmCherry* | Not I + Bgl II cut pNPC2-crmCherry |
| 2 | pNPC2-GSGSG-crmCherry | F1-NPC2; R3-5GS-NPC2; *pNPC2-crmCherry* | F3-5GS-NPC2; R2-mCherry; *pNPC2-crmCherry* | Not I + Bgl II cut pNPC2-crmCherry |
| 3 | pNPC2-(GS)5-crmCherry | F1-NPC2; R3-5GS-NPC2; *pNPC2-crmCherry* | F4-10GS-NPC2; R2-mCherry; *pNPC2-crmCherry* | Not I + Bgl II cut pNPC2-crmCherry |
| 4 | pLPO-crmCherry | Vector-F; Vector-R; *pNPC2-crmCherry* | LPO-F; LPO-R; *EST clone 40034365* | Nde I + Spe I cut pNPC2-crmCherry |
| 5 | pLPO-P5-crmCherry | F1-NPC2; R5-5P-LPO-L; *pLPO-crmCherry* | F5-5P-LPO-L; R2-mCherry; *pLPO-crmCherry* | Not I + Bgl II cut pNPC2-crmCherry |
| 6 | pLPO-P10-crmCherry | F1-NPC2; R5-5P-LPO-L; *pLPO-crmCherry* | F6-10P-LPO;R2-mCherry; *pLPO-crmCherry* | Not I + Bgl II cut pNPC2-crmCherry |
| 7 | pLPO-GSGSG-crmCherry | F1-NPC2; R7-5GS-LPO; *pLPO-crmCherry* | F3-5GS-NPC2; R2-mCherry; *pNPC2-crmCherry* | Not I + Bgl II cut pNPC2-crmCherry |
| 8 | pLPO-(GS)5-crmCherry | F1-NPC2; R7-5GS-LPO; *pLPO-crmCherry* | F4-10GS-NPC2; R2-mCherry; *pNPC2-crmCherry* | Not I + Bgl II cut pNPC2-crmCherry |
| 9 | pLPO-mCherry | F1-NPC2; R13-LPO-mCh; *pLPO-crmCherry* | F13-mCherry; R2-mCherry; *pLPO-crmCherry* | Not I + Bgl II cut pNPC2-crmCherry |
| 10 | pARSK-crmCherry | Vector-F; Vector-R; *pNPC2-crmCherry* | ARSK-F; ARSK-R; *EST clone 40146360* | Nde I + Spe I cut pNPC2-crmCherry |
| 11 | pARSK-P5-crmCherry | F1-NPC2; R8-5P-ARSK-L; *pARSK-crmCherry* | F9-5P-ARSK-L; R2-mCherry; *pARSK-crmCherry* | Not I + Bgl II cut pNPC2-crmCherry |
| 12 | pARSK-P10-crmCherry | F1-NPC2; R8-5P-ARSK-L; *pARSK-crmCherry* | F10-10P-ARSK-L; R2-mCherry; *pARSK-crmCherry* | Not I + Bgl II cut pNPC2-crmCherry |
| 13 | pARSK-GSGSG-crmCherry | F1-NPC2; R9-5GS-ARSK; *pARSK-crmCherry* | F3-5GS-NPC2; R2-mCherry; *pNPC2-crmCherry* | Not I + Bgl II cut pNPC2-crmCherry |
| 14 | pARSK-(GS)5-crmCherry | F1-NPC2; R9-5GS-ARSK; *pARSK-crmCherry* | F4-10GS-NPC2; R2-mCherry; *pNPC2-crmCherry* | Not I + Bgl II cut pNPC2-crmCherry |
| 15 | pARSK-mCherry | F1-NPC2; R14-ARSK-mCh; *pARSK-crmCherry* | F13-mCherry; R2-mCherry; *pLPO-crmCherry* | Not I + Bgl II cut pNPC2-crmCherry |
| 17 | pFUCA2-crmCherry | F1-NPC2; FUCA2-correct-R; *pFUCA2-crmCherry-mutation* | FUCA2-correct F; R2-mCherry; *pFUCA2-crmCherry-mutation* | Not I + Bgl II cut pNPC2-crmCherry |
| 18 | pFUCA2-P5-crmCherry | F1-NPC2; R11-5P-FUCA2-L; *pFUCA2-crmCherry* | F11-5P-FUCA2-L; R2-mCherry; *pFUCA2-crmCherry* | Not I + Bgl II cut pNPC2-crmCherry |
| 19 | pFUCA2-P10-crmCherry | F1-NPC2; R11-5P-FUCA2-L; *pFUCA2-crmCherry* | F12-10P-FUCA2-L; R2-mCherry; *pFUCA2-crmCherry* | Not I + Bgl II cut pNPC2-crmCherry |
| 20 | pFUCA2-GSGSG-crmCherry | F1-NPC2; R12-5GS-FUCA2; *pFUCA2-crmCherry* | F3-5GS-NPC2; R2-mCherry; *pNPC2-crmCherry* | Not I + Bgl II cut pNPC2-crmCherry |
| 21 | pFUCA2-(GS)5-crmCherry | F1-NPC2; R12-5GS-FUCA2; *pFUCA2-crmCherry* | F4-10GS-NPC2; R2-mCherry; *pNPC2-crmCherry* | Not I + Bgl II cut pNPC2-crmCherry |
| 22 | pFUCA2-mCherry | F1-NPC2; R15-FUCA2-mCh; *pFUCA2-crmCherry* | F4-10GS-NPC2; R2-mCherry; *pNPC2-crmCherry* | Not I + Bgl II cut pNPC2-crmCherry |
| 23 | pTPP1-crmCherry | Vector-F; Vector-R; *pNPC2-crmCherry* | Tpp1-mCherryF; Tpp1-mCherryR; *human cDNA library* | Nde I + Spe I cut pNPC2-crmCherry |
| 24 | pTPP1-P10-crmCherry | F1-NPC2; R-TPP1-10P; *pTPP1-crmCherry* | F-crmCherry; R2-mCherry; *pTPP1-crmCherry* | Not I + Bgl II cut pNPC2-crmCherry |
| 25 | pTPP1-(GS)5-crmCherry | F1-NPC2; R16-5GS-TPP1; *pTPP1-crmCherry* | F4-10GS-NPC2; R2-mCherry; *pNPC2-crmCherry* | Not I + Bgl II cut pNPC2-crmCherry |
| 29 | pCCL2-crmCherry | Vector-F; Vector-R; *pNPC2-crmCherry* | CCL2-F; CCL2-R; *human cDNA library* | Nde I + Spe I cut pNPC2-crmCherry |
| 30 | pCCL2-P10-crmCherry | Vector-F; Vector-R; *pNPC2-crmCherry* | CCL2-F; CCL2-10P-R; *human cDNA library* | Nde I + Spe I cut pNPC2-crmCherry |
| 31 | pCCL2-(GS)5-crmCherry | Vector-F; Vector-R; *pNPC2-crmCherry* | CCL2-F; CCL2-10GS-R; *human cDNA library* | Nde I + Spe I cut pNPC2-crmCherry |
| 32 | pOS9-crmCherry | Vector-F; Vector-R; *pNPC2-crmCherry* | OS9-F; OS9-R; *human cDNA library* | Nde I + Spe I cut pNPC2-crmCherry |
| 33 | pOS9-(GS)5-crmCherry | Vector-F; Vector-R; *pNPC2-crmCherry* | OS9-F; OS9-10GS-R; *human cDNA library* | Nde I + Spe I cut pNPC2-crmCherry |
| 34 | pNPC2 | F1-NPC2; R10-NPC2; *pNPC2-crmCherry* | N/A | Bgl II + Not I pNPC2-P5-crmCherry |
| 35 | pVector | N/A | N/A | Sma I + Hpa I cut pNPC2-P5-crmCherry |
| 36 | pNPC2-9aa-mCherry | pCMV-SPORT_F; NPC_ApaI_R; *EST clone 2649564* | N/A | Kpn I + Apa I cut pmCherry-N1 |
| 37 | pNPC2-5aa-mCherry | pCMV-SPORT_F; NPC_AgeI_R; *EST clone 2649564* | N/A | Kpn I + Age I cut pmCherry-N1 |
| 38 | pNPC2-5aa-mRFP1 | AgeI_K_mRFP1_F; NotI_mRFP1_R; *pAC-mRFP* | N/A | Age I + Not I cut pNPC2-5aa-mCherry |
| 39 | pNPC2-mCherry | pCMV-F; mNPC2-Spe-R; *pNPC2-5aa-mCherry* | mCh-Spe-F; pCMV-R; *pNPC2-5aa-mCherry* | EcoR V + Not I pNPC2-5aa-mCherry |
| 40 | pNPC2-crmCherry | pCMV-F; mNPC2-Spe-R; *pNPC2-5aa-mCherry* | mCh-Spe-F2; pCMV-R; *pNPC2-5aa-mCherry* | Spe I + Not I cut pNPC2-mCherry |
| 41 | pNPC2-P-crmCherry | pCMV-F; mNPC2-Spe-R; *pNPC2-5aa-mCherry* | 1Pro-mCh-F; pCMV-R; *pNPC2-5aa-mCherry* | Spe I + Not I cut pNPC2-mCherry |
| 42 | pNPC2-P2-crmCherry | pCMV-F; mNPC2-Spe-R; *pNPC2-5aa-mCherry* | 2Pro-mCh-F; pCMV-R; *pNPC2-5aa-mCherry* | Spe I + Not I cut pNPC2-mCherry |
| 43 | pNPC2-P3-crmCherry | pCMV-F; mNPC2-Spe-R; *pNPC2-5aa-mCherry* | 3Pro-mCh-F; pCMV-R; *pNPC2-5aa-mCherry* | Spe I + Not I cut pNPC2-mCherry |
| 44 | pNPC2-P4-crmCherry | pCMV-F; mNPC2-Spe-R; *pNPC2-5aa-mCherry* | 4Pro-mCh-F; pCMV-R; *pNPC2-5aa-mCherry* | Spe I + Not I cut pNPC2-mCherry |
| 45 | pNPC2-P5-crmCherry | pCMV-F; mNPC2-Spe-R; *pNPC2-5aa-mCherry* | 5Pro-mCh-F; pCMV-R; *pNPC2-5aa-mCherry* | Spe I + Not I cut pNPC2-mCherry |
| 47 | pTPP1 | TPP1-F; TPP1-R; *pTPP1-crmCherry* | N/A | Not I + Bgl II cut pNPC2-crmCherry |
