# Supplementary material for: Potential Pitfalls and Solutions for Use of Fluorescent Fusion Proteins to Study the Lysosome
Source: PLoS One. 2014 Feb 21;9(2):e88893. doi: 10.1371/journal.pone.0088893 (PMC3931630; doi:10.1371/journal.pone.0088893)
Supplement: Table S2 — Sequence of oligonucleotide primers used in construct synthesis. (DOCX) [file pone.0088893.s007.docx]

**Table S2: Sequence of oligonucleotide primers used in construct synthesis.**

| **Primer** | **Sequence 5’-3’** |
| --- | --- |
| F1-NPC2 | TACCGGACTCAGATCTCGAGCTCAAG |
| R1-10P-NPC2-L | ACTAGTGATCTGAACTGGGATCTC |
| F2-10P-NPC2-L | GTTCAGATCACTAGTCCGCCTCCCCCTCCTCCACCTCCACCTCCCATCATCAAGGAGTTCATGCGC |
| R2-mCherry | TCTAGAGTCGCGGCCGCTACTTGTAC |
| R3-5GS-NPC2 | GCCACTACCTGATCCACTAGTGATCTGAACTGGGATCTC |
| F3-5GS-NPC2 | GGATCAGGTAGTGGCATCATCAAGGAGTTCATGCGCTTC |
| F4-10GS-NPC2 | GGATCAGGTAGTGGCTCTGGGAGC GGATCG ATCATCAAGGAGTTCATGCGCTTC |
| Vector-F | AGTACATCAAGTGTATCATATG |
| Vector-R | CGTGGATATGAAAGCTCTGC |
| LPO-F | GCTTTCATATCCACGATGAGGGTCCTTCTCCATCT |
| LPO-R | GAACTCCTTGATGATATTCTTCACTGAGGCCCAGG |
| R5-5P-LPO-L | TGGATTCTTCACTGAGGCCCAGGGTG |
| F5-5P-LPO-L | TCAGTGAAGAATCCACCTCCGCCACCCATCATCAAGGAGTTCATGCGCTTC |
| F6-10P-LPO | TCAGTGAAGAATCCACCTCCGCCACCCCCTCCGCCACCACCCATCATCAAGGAGTTCATGCGCTTC |
| R7-5GS-LPO | GCCACTACCTGATCCATTCTTCACTGAGGCCCAGGGTG |
| R13-LPO-mCh | GCCCTTGCTCACCATATTCTTCACTGAGGCCCAGGG |
| F13-mCherry | ATGGTGAGCAAGGGCGAGGAG |
| ARSK-F | GCTTTCATATCCACGATGCTACTGCTGTGGGTGTC |
| ARSK-R | GAACTCCTTGATGATAACTGCTCTTGGATTCATAT |
| R8-5P-ARSK-L | TGGAACTGCTCTTGGATTCATATG |
| F9-5P-ARSK-L | CCAAGAGCAGTTCCACCTCCGCCACCCATCATCAAGGAGTTCATGCGCTTC |
| F10-10P-ARSK-L | CCAAGAGCAGTTCCACCTCCGCCACCCCCTCCGCCACCACCCATCATCAAGGAGTTCATGCGCTTC |
| R9-5GS-ARSK | GCCACTACCTGATCCAACTGCTCTTGGATTCATATGGG |
| R14-ARSK-mCh | GCCCTTGCTCACCATAACTGCTCTTGGATTCATATG |
| FUCA2-F | GCTTTCATATCCACGATGCGGCCCCAGGAGCTCCC |
| FUCA2-R | GAACTCCTTGATGATGATCACATTAGTCAGGGCTA |
| FUCA2-correct-R | TTCTCCATTGACTTTTAGCCAGGACCCCATTTGCCTCAGTCGCTCCTCAAAAAC |
| FUCA2-correct F | AAAGTCAATGGAGAAGCTATTTATGAAACCCATACCTGGCGATCCCAGAATG |
| R11-5P-FUCA2-L | TGGGATCACATTAGTCAGGGCTAGAG |
| F11-5P-FUCA2-L | ACTAATGTGATCCCACCTCCGCCACCCATCATCAAGGAGTTCATGCGCTTC |
| F12-10P-FUCA2-L | ACTAATGTGATCCCACCTCCGCCACCCCCTCCGCCACCACCCATCATCAAGGAGTTCATGCGCTTC |
| R12-5GS-FUCA2 | GCCACTACCTGATCCGATCACATTAGTCAGGGCTAGAG |
| R15-FUCA2-mCh | GCCCTTGCTCACCATGATCACATTAGTCAGGGCTAG |
| Tpp1-mCherryF | GCTTTCATATCCACGATGGGACTCCAA |
| Tpp1-mCherryR | GAACTCCTTGATGATGGGGTTGAGTAG |
| R-TPP1-10P | ATGAACTCCTTGATGATGGGTGGTGGCGGAGGGGGTGGCGGAGGTGGGGGGTTGAGTAGAGTCTTCAG |
| F-crmCherry | ATCATCAAGGAGTTCATGCG |
| R16-5GS-TPP1 | GCCACTACCTGATCCGGGGTTGAGTAGAGTCTTCAGC |
| RNASE6-F | GCTTTCATATCCACGATGGTGCTATGCTTTCCTCT |
| RNASE6-R | GAACTCCTTGATGATGAGAATACTATCTAAGTGTA |
| R-RNASE6-10P-2 | ATGAACTCCTTGATGATGGGTGGTGGCGGAGGGGGTGGCGGAGGTGGGAGAATACTATCTAAGTG |
| R17-5GS-RNASE6 | GCCACTACCTGATCCGAGAATACTATCTAAGTGTAC |
| CCL2-F | GAGCTTTCATATCCACGATGAAAGTCTCTGCCGCCCTTCTG |
| CCL2-R | ATGAACTCCTTGATGATAGTCTTCGGAGTTTGGGTTTGCTTG |
| CCL2-10P-R | ATGAACTCCTTGATGATGGGTGGTGGCGGAGGGGGTGGCGGAGGTGGAGTCTTCGGAGTTTGGGTTTGCTTG |
| CCL2-10GS-R | ATGAACTCCTTGATGATCGATCCGCTCCCAGAGCCACTACCTGATCCAGTCTTCGGAGTTTGGGTTTGCTTG |
| OS9-F | GAGCTTTCATATCCACGATGGCGGCGGAAACGCTGCTGTC |
| OS9-R | ATGAACTCCTTGATGATGAAGTCAAATTCGTCCAGGTCCC |
| OS9-10GS-R | ATGAACTCCTTGATGATCGATCCGCTCCCAGAGCCACTACCTGATCCGAAGTCAAATTCGTCCAGGTCCC |
| R10-NPC2 | TCTAGAGTCGCGGCCGCTATTAACTAGTGATCTGAACTGGGATC |
| NEU1-F | GCTTTCATATCCACGATGACTGGGGAGCGACCCAGCACG |
| NEU1-R | GAACTCCTTGATGATGAGTGTCCCATAGACACTGATTTTG |
| CREG1-F | GCTTTCATATCCACGATGGCCGGGCTATCCCGCGGGTCCG |
| CREG1-R | GAACTCCTTGATGATCTGAACTGTGACATTATAATATTC |
| ARSA-F | GCTTTCATATCCACGATGTCCATGGGGGCACCGCGGTCCC |
| ARSA-R | GAACTCCTTGATGATGGCATGGGGATCTGGGCAATGGCAG |
| CTSD-F | GCTTTCATATCCACGATGCAGCCCTCCAGCCTTCTGCCGC |
| CTSD-R | GAACTCCTTGATGATGAGGCGGGCAGCCTCGGCGAAGCCC |
| bGAL-F | GCTTTCATATCCACGATGCCGGGGTTCCTGGTTCGCATC |
| bGAL-R | GAACTCCTTGATGATTACATGGTCCAGCCATGAATCTTTG |
| CTSA-F | GCTTTCATATCCACGATGACTTCCAGTCCCCGGGCGCCTC |
| CTSA-R | GAACTCCTTGATGATGTATGGCTGCTTGTTCAGGAAGCGG |
| pCMV-SPORT_F | TAGGTGACACTATAGAAC |
| NPC_ApaI_R | TTTGGGCCCTGCTTGTGATCTGAAC |
| NPC_AgeI_R | TGACCGGTGGGCTTGTGATCTGAAC |
| AgeI_K_mRFP1_F | AAACCGGTCGCCACCATGGCCTCCTCCGAGGAC |
| NotI_mRFP1_R | ATTAAATGCGGCCGCTAGGCGCCGGTGGAGTG |
| pCMV-F | TGGTTTAGTGAACCGTCAG |
| mNPC2-Spe-R | ATTCTAGAGTCGCGGCCGCTACTTAATTACTAGTGATCTGAACTGGGATCTC |
| mCh-Spe-F | ATTCGTGGATATCTGACAAACTAGTATGGTGAGCAAGGGCGAGGAG |
| mCh-Spe-F2 | ATTCGTGGATATCTGACAAACTAGTATCATCAAGGAGTTCATGCGCTTC |
| pCMV-R | TAAAACCTCTACAAATGTG |
| 1Pro-mCh-F | GATATCTGACAAACTAGTCCCATCATCAAGGAGTTCATG |
| 2Pro-mCh-F | GATATCTGACAAACTAGTCCTCCCATCATCAAGGAGTTCATG |
| 3Pro-mCh-F | GATATCTGACAAACTAGTCCACCTCCCATCATCAAGGAGTTCATG |
| 4Pro-mCh-F | GATATCTGACAAACTAGTCCACCTCCACCCATCATCAAGGAGTTCATG |
| 5Pro-mCh-F | GATATCTGACAAACTAGTCCACCTCCACCTCCCATCATCAAGGAGTTCATG |
| TPP1-F | CTAGCGCTACCGGACTCAGATC |
| TPP1-R | ATGCATTCTAGAGTCGCGGCCGCTAGGGGTTG |
